# Understanding the Role of Deconjugation of Phase II Metabolites in Wastewater: Implications for Wastewater-Based Epidemiology

**DOI:** 10.1021/acs.est.5c17466

**Published:** 2026-02-09

**Authors:** Harry Elliss, Katarina Hricova, Evie Griffiths, Neil Andrew Byrnes, Ben Faill, Eva Hawkins, Kit Proctor, Megan Robertson, John Bagnall, Barbara Kasprzyk-Hordern

**Affiliations:** † Department of Chemistry, 1555 University of Bath, Claverton Down, Bath BA2 7AY, U.K.; ‡ Centre of Excellence in Water-Based Early-Warning Systems for Health Protection, 329237 University of Bath, Claverton Down, Bath BA2 7AY, U.K.; § Institute of Sustainability and Climate Change, University of Bath, Claverton Down, Bath BA2 7AY, U.K.; ∥ Australian Centre for Research on Separation Science, School of Chemistry, Monash University, Wellington Road, Clayton, Victoria 3800, Australia; ⊥ Chemical Characterisation Facility, University of Bath, Claverton Down, Bath BA2 7AY, U.K.; # Wessex Water Service Ltd., Claverton Down, Bath BA2 7WW, U.K.

**Keywords:** wastewater-based epidemiology, stability, glucuronide, sulfate, enzymatic deconjugation, phase II metabolism

## Abstract

Metabolism is a critical bodily function that facilitates the removal of toxic chemical buildup within the body. In wastewater-based epidemiology (WBE), it is crucial to understand the metabolism of biochemical indicators (BCIs) because metabolites are indicative of consumption (e.g., illicit drugs, pharmaceuticals) or unintentional exposure (e.g., pesticides, endocrine disruptors). Phase I metabolites are more widely studied in WBE due to a combination of factors, including, but not limited to, stability and analyte cost. Phase II metabolites are often assumed to deconjugate within the sewer network due to high native concentrations of enzymes. This work deconstructs this assumption and demonstrates how the in-sewer stability of phase II metabolites is dependent on both the parent structure and the conjugate type. In total, 79 BCIs were assessed and compared to urinary metabolism studies via time-variable enzymatic deconjugation using two enzymes, β-glucuronidase and arylsulfatase. The concentrations of free analytes excreted as N-glucuronides, O-glucuronides, and sulfates increased following deconjugation, reinforcing the persistence of these BCIs during transport throughout the sewer network. Conversely, no concentration increase was observed for acylglucuronides, demonstrating complete in-sewer glucuronide cleavage. In-freezer stability of conjugates was also assessed over 6 months, where it was observed that the stability of the parent structure is the driver of stability rather than the conjugates themselves, indicating minimal enzymatic activity upon storage. Overall, this paper presents a framework that can be deployed to gain a more comprehensive understanding of phase II metabolism and improve the accuracy of WBE workflows as well as environmental risk assessment approaches.

## Graphical abstract

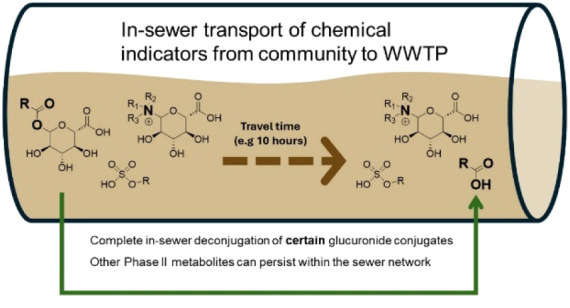

## 1 Introduction

Wastewater-based epidemiology (WBE) has been used to establish a comprehensive understanding of the consumption or exposure to various chemical classes, providing great insights into community health. Numerous chemical classes have been assessed, including lifestyle chemicals, such as licit and illicit drugs;^1–8^ pharmaceuticals,^9^ such as painkillers,^10,11^ antibiotics,^12^ or antidepressants;^13,14^ and food.^15–18^ Alongside intended consumption, WBE is also used to estimate community-level exposure to hazardous chemicals such as pesticides,^19^ bisphenols,^20^ or flame retardants.^21^ Due to the uncontrolled nature of untreated wastewater, often coming from multiple sources such as trade and human waste, there are critical requirements for selecting a suitable biomarker for analysis. A review by Gracia-Lor et al.^22^ demonstrates the importance of several characteristics, including stability and the marker originating from a unique source following human metabolism. There are two main metabolism routes: phase I, where there is a chemical change, often via oxidation, using Cytochrome P450 enzymes, and phase II, where a conjugate is added to the parent structure; both routes aim to increase hydrophilicity and aid excretion.^23^ There are multiple types of conjugates for phase II metabolism, such as acyl, sulfate, and glucuronide, with glucuronides, formed via glucuronidation, being the most common.^24–26^ Typically, phase II metabolism is facilitated by transferase enzymes, where UDP-glucuronosyltransferases (UGTs), sulfotransferases (SULTs), and glutathione S-transferases (GSTs) are the most common for glucuronidation, sulfation, and glutathione conjugation, respectively.^27^

In WBE back-calculations, phase I metabolites are preferred due to the cost and availability of the analytical standard, enabling full quantification. In these back-calculations, it is often assumed that complete glucuronide deconjugation occurs.^11,28–32^ Back-calculations are typically performed by developing correction factors based on human excretion (%) obtained from human biomonitoring or pharmacokinetic studies.^9,19^ Recent approaches have developed new correction factors based on WBE data by comparing experimental data to other sources of data, such as product sales or pharmaceutical prescriptions.^33,34^ While the recent approaches to develop new correction factors have improved the accuracy of back-calculation, it is critical to fully understand in-sewer processes such as deconjugation, which may vary in different sewer networks across the world.^33,34^ Improving an understanding of the impact of phase II metabolism within sewer systems will enable the WBE research field to better understand possible areas of variability.

In-wastewater stability assessments for some phase II metabolites have been conducted, including ethyl sulfate and ethyl glucuronide, the phase II metabolites of alcohol, and cotinine-N-β-glucuronide and trans-3′-hydroxycotinine-O-β-glucuronide, the phase II metabolites of cotinine.^35–37^ While rapid degradation of ethyl glucuronide and trans-3′-hydroxycotinine-O-β-glucuronide was observed,^35,37^ degradation at a lower rate was observed for ethyl sulfate (8% degradation per hour in a real rising main^35^) and cotinine-N-β-glucuronide (less than 20% degradation at room temperature after 7 days^37^). This indicates that some phase II metabolites undergo rapid degradation in wastewater due to alternative sources of these enzymes, such as *E. coli*.^38^ However, this is not the case for all metabolites, requiring further investigation to fully understand the impact of the phase II metabolism on future WBE frameworks.

Deconjugating phase II metabolites enables an accurate and complete understanding of human metabolism for WBE and is critical to its successful implementation. Several wastewater studies have utilized β-glucuronidase enzymes during sample preparation, most popularly for the detection of oxidative stress biomarkers, isoprostaglandins,^39–42^ where increased detectability is observed following enzymatic deconjugation. Following deconjugation, an enhanced signal reinforces the importance of understanding phase II metabolism, as here, glucuronide conjugates do not fully degrade in the sewer system.^39–42^ Alongside alcohol, nicotine, and isoprostaglandins, glucuronide conjugates of other BCIs have been investigated in wastewater, including pharmaceuticals, such as carbamazepine, thyroxine, and lamotrigine;^43–46^ benzodiazepines (via deconjugation);^41^ and opioids.^47^ Alongside glucuronidation, sulfated conjugates are also present in wastewater (e.g., acetaminophen^48^ and estrogenic hormones^28,49^ ). The β-glucuronidase enzyme, originating from *E. coli*, is present in high concentrations in the sewer system;^38^ however, its limited arylsulfatase activity limits in-sewer degradation, potentially explaining the persistence of sulfate conjugates.^50^

During urine analysis, a common sample preparation technique is enzymatic deconjugation with both β-glucuronidase and arylsulfatase enzymes to analyze the free, unconjugated compound.^51,52^ This manuscript aims to utilize both β-glucuronidase and arylsulfatase enzymes to further investigate the impact of phase II metabolism on 79 common chemical markers frequently analyzed in WBE across a range of subclasses.

## 2 Materials and Methods

### 2.1 Reagents and Analytical Standards

Analyte and isotopically labeled standards (>98%) were purchased from Merck (Gillingham, UK), LGC (Teddington, UK), and Cambridge Bioscience (Cambridge, UK). The full list of analytes and internal standards used in this method is reported in Table S1. Analyte standards covering five biomarker classes (pharmaceuticals, food, endogenous, lifestyle, and personal care products (PCPs)) were purchased either as methanolic solution or as powder and made to 1.0 mg mL^–1^. Riboflavin and 4-pyridoxic acid were prepared to 0.1 mgmL^–1^ due to the low solubility. Isotopically labeled standards were purchased at either 1.0 mgmL^–1^ or 0.1 mgmL^–1^, depending on the available concentrations. Methanol and water (LC-MS grade) were purchased from Merck. Mobile phase additives, formic acid, ammonium formate, and ammonium fluoride, were purchased from Merck. The enzymes used for deconjugation were β-glucuronidase (from *Helix pomatia*, type H-2, ≥85,000 units/mL) and arylsulfatase (from *Helix pomatia*, type H-1, ≥10,000 units/g), and both were purchased from Merck. Arylsulfatase was solubilized in 100 mM ammonium acetate, adjusted to pH 5.0 with acetic acid. Both acetic acid and ammonium acetate (>95%) were purchased from Merck.

A primary aim of this manuscript is to demonstrate the impact of phase II metabolism on a broad range of chemical classes frequently analyzed within WBE. Table 1 provides a summary of the analytes under study, while Tables S1 and S2 provide greater detail on the analytes, including supplier information and the types of phase II conjugation, respectively. Some phase II metabolites, such as indoxyl sulfate, were also selected for analysis as a control to ensure that the enzymatic deconjugation had been successful, where a decrease in the concentration of the observed sulfate-conjugate was seen over time.

**Table 1:** Analyte Class Breakdown for Target Molecules within This Manuscript^a^

Analyte class	Number of compounds	Compound breakdown
Illicit drugs	8	5 parent compounds
		3 phase I metabolites
Lifestyle	4	2 parent compounds
		2 phase I metabolites
Pharmaceuticals	44	30 parent compounds
		13 phase I metabolites
		1 phase II metabolite
Endogenous human markers	9	1 phase II metabolite
		8 unconjugated human metabolites
Food	11	4 parent compounds
		6 phase I metabolites
		1 phase II metabolite
PCPs	3	3 parent compounds

a A further breakdown is provided in Tables S1 and S2.

Literature reports, summarized in Table S2, highlight that target analytes can form numerous phase II conjugates, each with its own excretion rate. The relative proportion of phase II conjugates identified in these works is summarized in Figure 1. As the vast majority of targets that contain phase II metabolites form either sulfate or glucuronide conjugates, these will be the main focus of this study. Other phase II metabolites, such as taurine or glycine conjugates, warrant future research.

**Figure 1:**
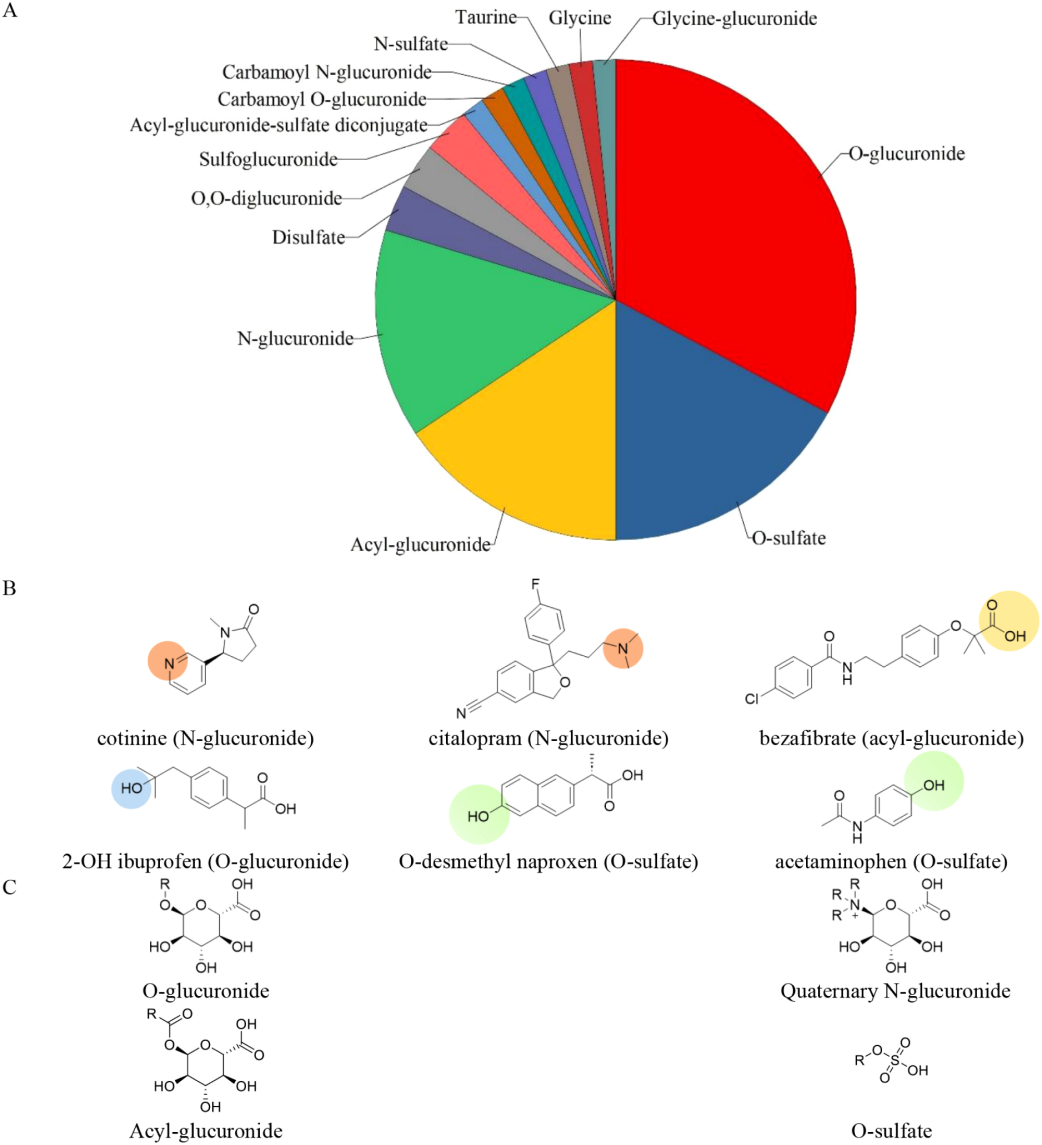
(A) Breakdown of the conjugation types of phase II metabolites under study. (B) Analyte examples with a demonstration of the most common conjugation types within this manuscript. Additional structures are shown in Figures S1–S5 within the Supporting Information. (C) Example structures of the conjugate, where R indicates the chemical that has undergone phase II metabolism.

This article covers a broad range of targets, which also undergo different mechanisms of conjugation, such as mercapturic acid and acetylcysteine (HNE-MA and D,L-sulforaphane-N-acetylcysteine, respectively).

### 2.2 Monitoring Deconjugation over Time

Untreated wastewater (from a city with 1.2% industrial input) was collected after physical screening. The sewer network under study includes a 7.2 km rising main and overall has a hydraulic retention time from 6 to 27 h (depending on rainfall). Upon collection, samples were transported on ice from the wastewater treatment plant (WWTP) to the laboratory to prevent biological activity. All wastewater was filtered (GF/F 0.7 μm, Whatman) and pooled (2.6 L) to ensure a homogeneous sample. Upon pooling, the wastewater was adjusted to pH 5.0 to ensure maximum enzymatic activity and, later, split into 100 mL aliquots. A T_0_ aliquot was taken and prepared via solid-phase extraction (SPE).

The effect of different incubation times was monitored, with 7 time points across 30 min to 18 h. To ensure a good starting point, the times and concentrations were compared to those in other enzymatic deconjugation experiments performed in wastewater and urine (see Table S3). Table 2 shows the incubation times and enzyme concentrations employed. Enzymes from *Helix pomatia* were used, which have been widely used in the literature for deconjugation in wastewater, as displayed in Table S3. Commercially available β-glucuronidase and arylsulfatase from *Helix pomatia*, both have activity toward the other enzyme. Therefore, to fully understand enzyme function, an equal activity of β-glucuronidase was added in both incubation vials with differing arylsulfatase activity.

**Table 2:** Time and Enzyme Concentrations for Sample Incubation during Enzymatic Deconjugation^a^

	β-glucuronidase, type H-2 from *Helix pomatia*		Arylsulfatase, type H-1 from *Helix pomatia*	
Time/h	β-glucuronidase concentration (units/mL)	Arylsulfatase concentration (units/mL)	β-glucuronidase concentration (units/mL)	Arylsulfatase concentration (units/mL)
0	n/a	n/a	n/a	n/a
0.5	40,000	267	40,000	1400
1	40,000	267	40,000	1400
1.5	40,000	267	40,000	1400
3	40,000	267	40,000	1400
5	40,000	267	40,000	1400
18	40,000	267	40,000	1400

a All incubation was conducted at 37 °C.

To ensure observed concentration changes were due to enzymatic activity, each time point had a control. Here, the control sample was taken from the original pooled sample and incubated for all time points above. The control is critical to the validity of this work, as it is possible that any concentration change may occur due to transformation within the matrix at elevated temperatures to those typically observed in sewer networks. Following incubation, each sample was left to cool to room temperature, adjusted to pH 7.0 with 1 M ammonium hydroxide, and spiked with 100 μL of a 1 μg mL^–1^ mix of isotopically labeled standards to enable quantification.

### 2.3 Sample Preparation—In-Freezer Wastewater Stability of Phase II Metabolites

Following the deconjugation optimization outlined in Section 2.2, the 3-h time point was selected to provide an understanding of BCI deconjugation with a single time point, reducing sample processing and cost. Often in WBE, it is not possible to immediately process samples from the WWTP; therefore, an understanding of sample stability in the freezer is critical to BCI validity.^9^ Two liters of influent wastewater were pooled, split into 100 mL aliquots, and spiked with 100 μL of a 1 μg mL^–1^ mix of isotopically labeled standards prior to freezing at −28 °C. Five time points, spread across 6 months, were used to assess analyte stability: 7, 15, 35, 120, and 180 days. At each time point, the three samples were defrosted, pH adjusted, and (1) no enzyme added, (2) spiked with β-glucuronidase, and (3) spiked with arylsulfatase, then incubated as per Section 2.2.

### 2.4 Sample Preparation—Bulk Wastewater Stability of Free Analytes

Similar to Section 2.3, it is important to assess the wastewater stability at room temperature, often reflective of the stability during in-sewer transport. Influent wastewater was pooled, split into 100 mL aliquots, and stored at room temperature for 7 different time points spread across 24 h (1 h, 3 h, 5 h, 8 h, 16 h, 20 h, 24 h). To reduce the impact of adding methanol and disrupting native microbial processes in the wastewater, analytes were not spiked into the samples. As a result, some targets were not detected.

### 2.5 Solid-Phase Extraction

Following enzyme incubation, pH adjustment, and spiking with isotopically labeled standards, the samples were split into 50 mL replicates for solid-phase extraction as per a previously validated method.^53^

### 2.6 Instrumentation

The analytes were separated using reverse-phase liquid chromatography, using a BEH C18 column (150 mm × 1.0 mm, 1.7 μm) fitted with a 0.2 μm, 2.1 mm in-line column filter (Waters, Manchester, UK). Identification and quantification of analytes were performed using the Xevo TQD Triple Quadrupole Mass Spectrometer (Waters, Manchester, UK), equipped with an electrospray ionization (ESI) source. Two LC-MS/MS methods were used to quantify analytes: method A using the ESI source in positive mode, and method B using negative mode. Details of the ESI+ and ESI- methods are reported in the previously validated method.^53^ For the qualitative analysis of 8-isoprostaglandin F2_a/β_, an alternative method was used.^54^ For all samples, data processing was performed using MassLynx (version 4.1, Waters) and TargetLynx software (Waters).

### 2.7 Calculations

The extent of conjugation was understood via the comparison of calculated concentrations and peak responses across different enzyme conditions and time points. Response and concentration were calculated using eqs 1 and 2 below.

1 $$\mathrm{Response}=\mathrm{analyte peak area}\times \left(\frac{\mathrm{Internal standard concentration}}{\mathrm{Internal standard peak area}}\right)$$

2 $$\mathrm{Concentration}\left(ng/L\right)=\left(\frac{response-c}{m}\right)\times \frac{1000}{cf}$$

where *m* and *c* are the gradient and intercept of the calibration curve, and cf is the concentration factor during SPE.

## 3 Results and Discussion

### 3.1 Estimating the Matrix Suppression Following Enzyme Addition

It was not possible to have matching isotopically labeled standards for all 79 analytes. To ensure the validity of results, in the cases where matching labeled standards were not possible, the degree of signal suppression for labeled standards used was calculated for both enzymes and normalized to the control. This suppression was estimated simply by the change in the area under the curve (AUC) to estimate the significance of observed concentration changes. Across the 27 labeled standards, the average % peak area (normalized to the control AUC) for samples containing β-glucuronidase and arylsulfatase was 85.6% ± 40.3% and 102.2% ± 43.7%, respectively. It is important to note that these values were obtained using the methods detailed above in Section 2, and it is likely that observed suppression would vary depending on the analytical workflow deployed. Literature studying transformations (stability) in wastewater has typically used a combination of <10% or <20% concentration change over time as indicative of good stability.^55–57^ Due to the suppression range above, an observed change in concentration of >20%, when compared to the maximum concentration change of the control sample, was deemed significant. This method accounts for in-wastewater transformation at 37 °C not due to enzyme addition. Due to broad suppression ranges, it is possible that observed changes are due to analytical variability; however, by selecting the more conservative value to demonstrate significance, this possibility is reduced.

### 3.2 Monitoring Enzymatic Deconjugation over Time

To ensure complete deconjugation, the concentration change over multiple time points was monitored as outlined in Table 2. Here, we observed four key scenarios: (i) an increase in the concentration of the free analyte, (ii) a decrease in the concentration of a phase II conjugate, (iii) no concentration change observed, and (iv) degradation of the free analyte due to enzyme addition.

#### 3.2.1 Concentration Change of Free Analytes Following Enzymatic Deconjugation

Per the criteria outlined in Section 3.1, 43 of the 79 compounds observed a concentration increase, and 22 of the 46 increased with both enzymes. This provides additional confirmation of the deconjugation of phase II metabolites. Further discussion throughout this manuscript will focus on the deconjugation within only one of the enzymes. These results, detailing the increase in concentration of the free analyte following enzymatic deconjugation, are captured in Table S4 and Figures S6–S18. This increase affecting a broad range of compounds (ranging from mirtazapine, a tetracyclic antidepressant, to enterolactone, a dietary metabolite of lignan), analyzed via typical targeted methods, could impact the accuracy of WBE back-calculations if phase II metabolism is not accounted for. As mentioned in the introduction, the phase II metabolite of cotinine, cotinine-N-β-glucuronide, has previously been detected in wastewater with moderate stability.^37^ Here, we observed that both β-glucuronidase and arylsulfatase (with equal β-glucuronidase activity, as per Figure 2A) rapidly deconjugate cotinine-N-β-glucuronide, reaching equilibrium at a similar rate.

**Figure 2:**
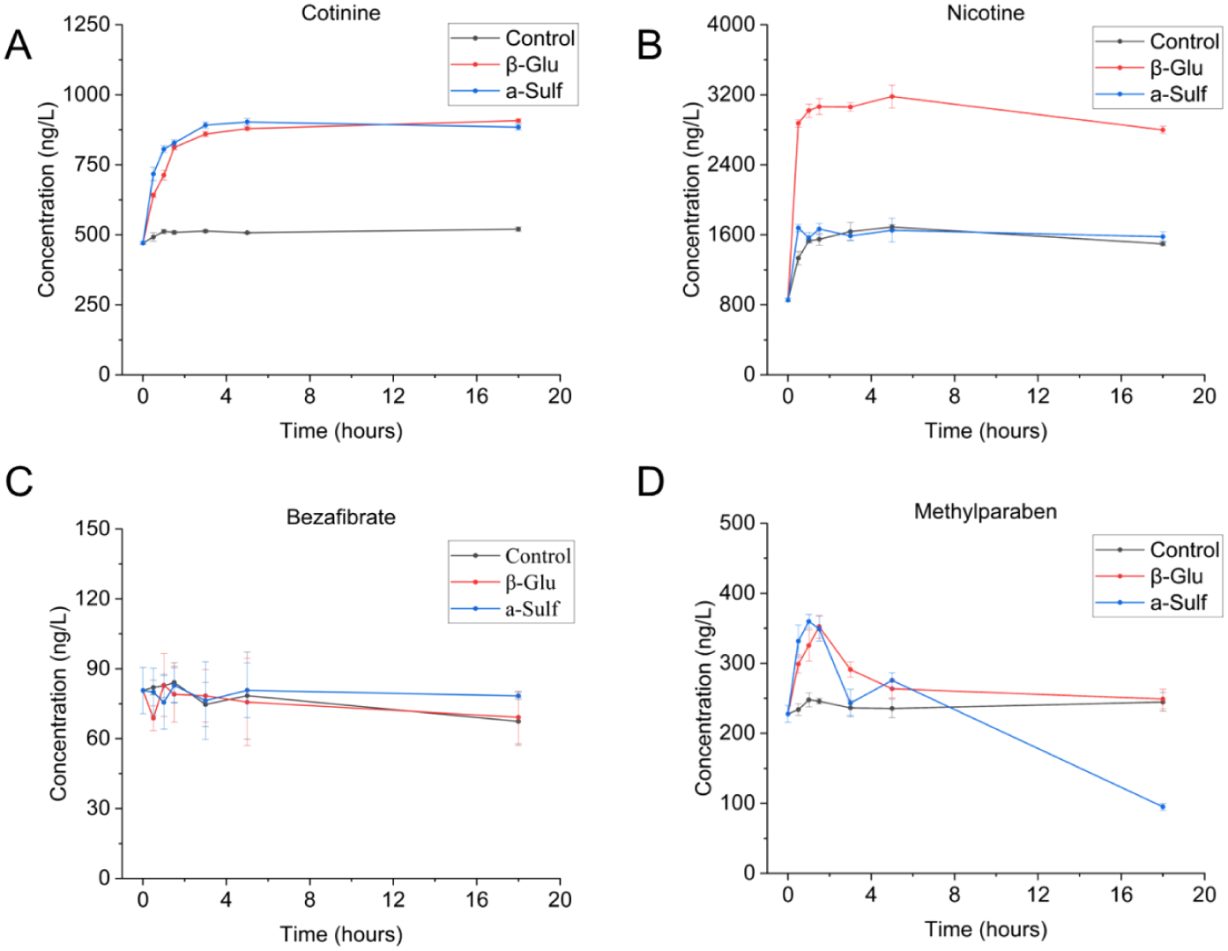
Time–concentration plot of the enzymatic deconjugation of (A) cotinine, (B) nicotine, (C) bezafibrate, and (D) methylparaben (where β-Glu is the sample that has been incubated with β-Glucuronidase enzyme and a-Sulf has been incubated with arylsulfatase enzyme).

There was no significant difference between the final (18 h) concentrations of cotinine in wastewater samples incubated with β-glucuronidase and arylsulfatase, where a concentration increase of 92.8% ± 0.8% and 91.8% ± 1.4%, respectively, was observed when compared to T_0_. During the analysis of nicotine (Figure 2B), only an increase in the concentration of free analyte was observed following deconjugation with β-glucuronidase. While an explanation could simply be due to observed suppression (Section 3.1), it is also possible that the two enzymes have differing activity for certain compounds. Other analytes, which also followed the same phenomena either due to deconjugation or other transformation processes, include amphetamine, 5-hydroxy lansoprazole, and desmethyl citalopram. This is discussed further in Section 3.4.

Crucially for current sample preparation approaches, an increased concentration following deconjugation was not observed for a vast number of compounds analyzed, highlighting the validity of current approaches. An example of this is captured in Figure 2C, where bezafibrate shows no concentration change over time. Other such analytes include benzoylecgonine, ketamine, cocaine, MDMA, saccharin, methadone, dihydrocodeine, and cocaethylene. A summary of all analytes is displayed below in Figure 5. In the above scenarios, where no change in the concentration of free analyte was observed, other analytes that require deconjugation can be analyzed in parallel. This increases the breadth of targets able to be analyzed simultaneously, limiting time spent on sample preparation, as one method can be used for a wide variety of classes. This is often not used in the analysis of wastewater when performing enzymatic deconjugation.^41^

Alongside an increase in the concentration of the free analyte after enzyme treatment, it was also observed that some compounds underwent degradation. One example of this is methylparaben (Figure 2D), where a rapid initial increase was observed, indicative of deconjugation and therefore human exposure to methylparaben. Both enzymes reached similar maxima and then decreased in concentration at different rates. This has also been previously observed in urine analysis of methylparaben, where it was hypothesized that this was due to alkyl/aryl enzymatic hydrolysis of the alkyl-ester bond within methylparaben.^50,58,59^ This was also seen in phthalate diesters, due to the same functional group, again associated with the lipase activity inherent within commercially available sulfatase enzymes.^60^ This degradation was also observed in several compounds that also contain primary amide functional groups, such as levetiracetam and phenylacetylglutamine, therefore requiring additional steps to be taken during sample processing to mitigate future uncertainty. Recent work in human biomonitoring has demonstrated the importance of enzyme source and the incompatibility of *Helix pomatia* enzymes where there is a possibility of enzymatic breakdown of compounds, which would lead to overestimation as product formation is not due to human metabolism.^58^ It is important to note that commercially available enzyme mixtures used within this work (H-1 and H-2) can contain secondary activities such as esterases and lipases.^61^ Furthermore, *Helix pomatia* is known to have deaminase activity; therefore, it is possible that this could have an impact on other analytes.^62^ To limit side reactions and enable a true understanding of phase II metabolites, future work should look to explore the purification of enzymes or recombinant enzymes to increase the rate of deconjugation and limit uncertainty in possible side reactions.^50,58,63–65^

### 3.3 Simultaneous Concentration Change: Increased Concentration of Free Analyte and Decreased Concentration of the Phase II Metabolite Following Enzymatic Deconjugation

A comprehensive understanding of the fate of phase II metabolites in wastewater can be achieved by simultaneously monitoring a parent compound and its phase II metabolite. Here, acetaminophen and acetaminophen sulfate were used to demonstrate this. It has been previously observed that acetaminophen glucuronide is not detected in samples collected from WWTPs;^66^ to our knowledge, it has only been detected during near-source sampling from a manhole.^47^ Due to large proportions of acetaminophen glucuronide excreted in urine^67^ this indicates it is highly unstable in wastewater. Figure 3 focuses on the deconjugation of acetaminophen sulfate to acetaminophen.

**Figure 3:**
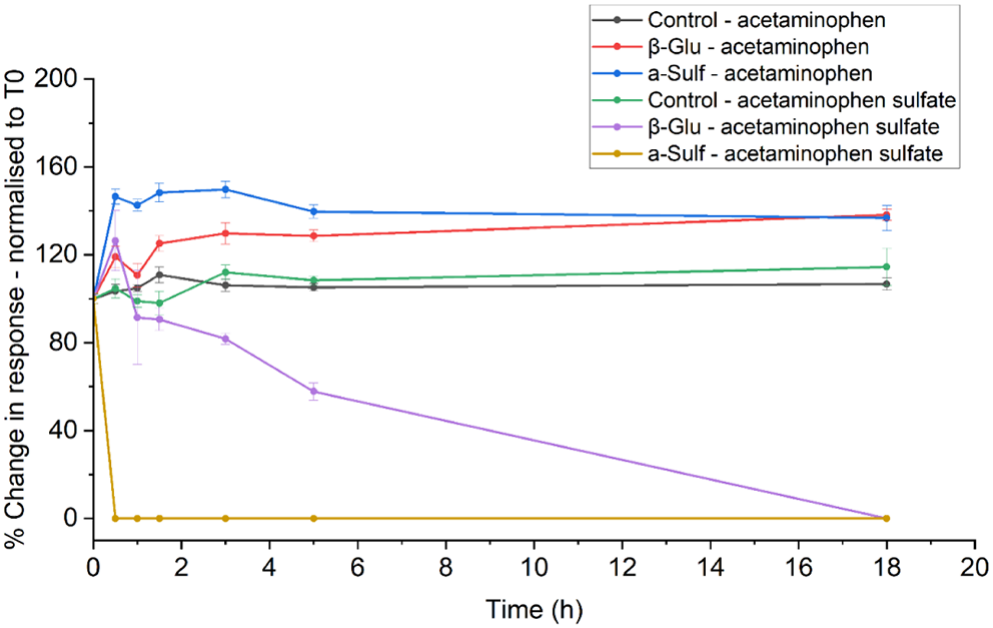
Time-recovery plot of the enzymatic deconjugation of acetaminophen sulfate to acetaminophen (where β-Glu is the sample that has been incubated with β-Glucuronidase enzyme and a-Sulf has been incubated with arylsulfatase enzyme).

Figure 3 confirms this transformation, where the deconjugation of sulfate conjugates is dependent on enzymatic activity; a rapid increase in acetaminophen (combined with a rapid decrease in acetaminophen sulfate) is observed when using the arylsulfatase enzyme. Using this data, it can be predicted via calculations of acetaminophen concentration that the initial concentration of acetaminophen sulfate was 22 μg/L, 28% of the total free acetaminophen after deconjugation. This value aligns closely with the urinary excretion rate of 30–44% reported in the literature.^68^ Alongside acetaminophen sulfate, the same observations were observed with indoxyl sulfate, a dietary metabolite of tryptophan. Again, a rapid decrease in the concentration of indoxyl sulfate was observed with arylsulfatase when compared to β-glucuronidase (see Figure S14).

### 3.4 Deconjugation with Different Enzymes Reaches Different Equilibria

In this research, two enzyme types were used, both from *Helix pomatia*: type H-1 and type H-2. As the arylsulfatase and β-glucuronidase enzymes each display activity for both sulfate and glucuronide conjugates, the type H-1 arylsulfatase was made into a solution to obtain an activity equal to that of the type H-2 β-glucuronidase. Different rates of deconjugation of sulfate conjugates are therefore expected due to the differing arylsulfatase enzyme activity. Despite this, multiple observations of differing β-glucuronidase activity were observed between the type H-1 and type H-2 enzymes. One key example is with 8-isoprostaglandin F2_a/β_. When samples were treated with type H-2 enzyme, clear peaks emerged on the chromatogram consistent with 8-isoprostaglandin F2_a/β_, even though it was not observed in T_0_ or the control, in line with numerous literature reports.^39–42^ However, there was no observed glucuronide deconjugation (indicated by no peak, Figure S19) during samples treated with the H-1 arylsulfatase enzyme. This discrepancy between type H-1 and type H-2, hypothesized to be due to incomplete deconjugation, has been previously documented by Dwividi et al., where the H-1 enzyme fully deconjugated triclocarban N-glucuronide while the HP-2 enzyme did not.^50^ It is also important to consider that the enzyme structure may have prevented deconjugation for some of the 22 analytes, where an increase in concentration was observed with only one enzyme. Additionally, it is possible that this could be due to increased suppression when using one enzyme compared to the other. The same rate-dependent observations were seen for many compounds (e.g., mirtazapine and morphine in Figure 4). This could be due to multiple reasons, such as the analytes’ structure or the preparation method of the enzyme, purchased as a powder (H-1) or in solution (H-2).

**Figure 4:**
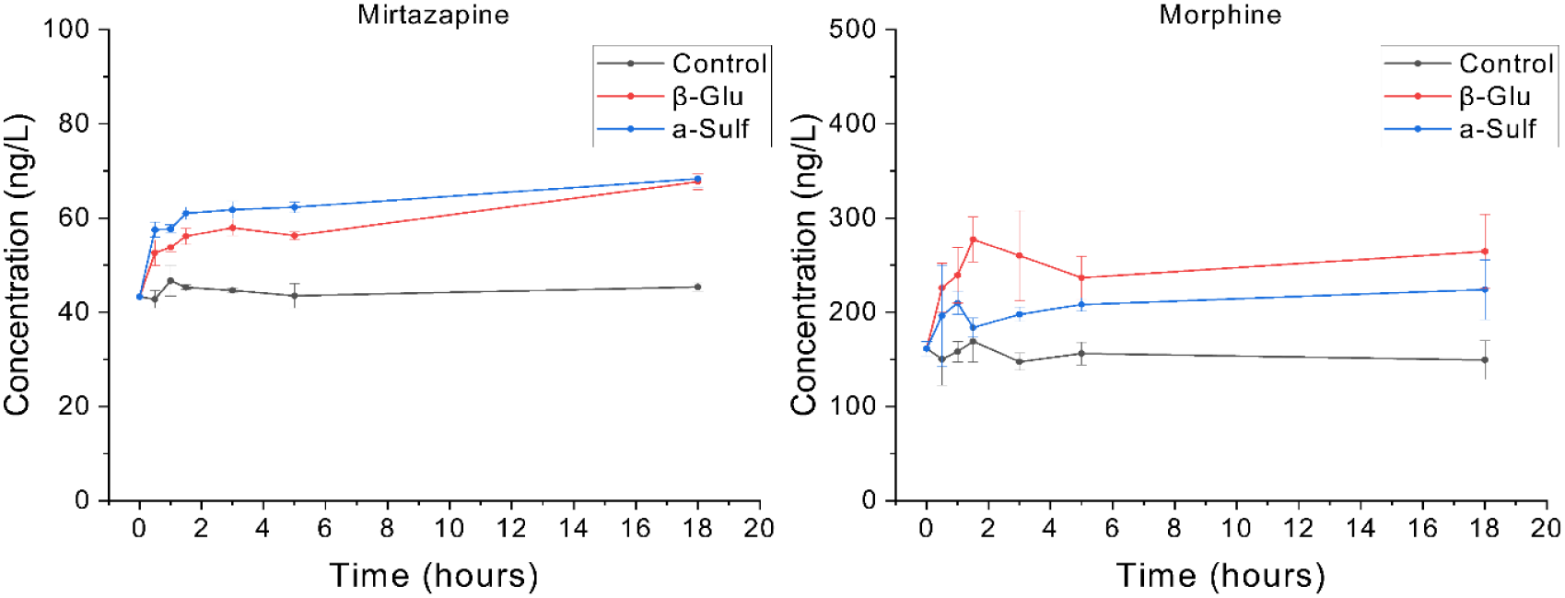
Time–concentration plot of the enzymatic deconjugation of mirtazapine (left) and morphine (right) (where β-Glu is the sample that has been incubated with β-Glucuronidase enzyme and a-Sulf has been incubated with arylsulfatase enzyme).

Mirtazapine N-glucuronide is the phase II metabolite of mirtazapine. Upon addition of enzyme to the sample, the H-1 arylsulfatase enzyme undergoes more rapid deconjugation than the H-2 β-glucuronidase, but critically, the extent of deconjugation is not affected. This demonstrates the importance of assessing incubation time during method development to ensure complete deconjugation. The extent of deconjugation is not always the same between enzymes, and this is evident with morphine, where the H-2 arylsulfatase frees more of the parent compound. This relationship occurs for a variety of compounds, both N- and O-glucuronides, indicating the importance of time-variable studies. Future studies in wastewater should deploy kinetic work to evaluate the kinetic parameters of each analyte and identify structural links that may cause changes in the rate of deconjugation, gaining a further understanding of chemicals that may be more likely to deconjugate within the sewer network.^69^ To ensure full and accurate quantification of the extent of phase II metabolites in wastewater, sample preparation must be optimized in an analyte-dependent manner. Most analytes did reach equilibrium, as indicated by Mann-Kendall analysis, which highlights if a trace is facing an upward or downward trend (Table S5).

### 3.5 Total Observed Deconjugation, Implications for WBE

As discussed in Sections 3.2–3.4, there is a broad range of % concentration increases across five different subgroups (pharmaceuticals, food, PCPs, and endogenous and lifestyle chemicals). This indicates the impact of phase II metabolism on current targeted analytical methods. To understand the total observed deconjugation, a range is given. Here, the maximum deconjugation is indicated by the comparison of the largest concentration change, upon enzyme addition, compared to T_0_. The minimum deconjugation is indicated by the comparison of the largest concentration change upon enzyme addition compared to the highest concentration of the control. This would account for any potential changes due to transformation products.

Figure 5 indicates that the extent of phase II metabolism varies per compound, as expected, and potential variation in analyses due to the compounds’ variable stability must be accounted for. The amount of deconjugation observed (via the increase of the free analyte) appeared to vary depending on the type and location of the glucuronide conjugate (Figures S1–S5). Acyl conjugates are observed in urinary analysis for the seven compounds under study (Table S3). It is widely reported that these conjugates are highly reactive due to the electron-deficient carbonyl center, making them susceptible to both alkaline and enzymatic hydrolysis.^70^ On the other hand, N-glucuronides have enhanced stability in wastewater, which we hypothesized is due to the lower reactivity of these conjugates. In the case of quaternary conjugates (e.g., cotinine and nicotine), the resonance-stabilized charge reduces the reactivity, whereas in the case of carbamide tertiary conjugates (e.g., carbamazepine), the charge is also able to resonate, increasing the stability and persistence of these chemicals. It is hypothesized that these factors cause increased stability over O-conjugates. This was further investigated in context with known urinary excretion rates of phase II metabolites (Table S2). Here, conjugates with a known excretion rate were compared to the urinary excretion of phase II metabolites (excluding mirtazapine, quetiapine, and tramadol, where the excretion rate is < *x*, where *x* is 1 for mirtazapine and quetiapine and *x* = 2 for tramadol, and enterolactone and enterodiol, as they both have five different conjugates). A limitation of this method is that, when analyzing the free analyte after enzymatic deconjugation, it is not possible to determine which conjugate had persisted. In the case of enterolactone and enterodiol, due to an increased concentration change following arylsulfatase deconjugation, it is expected that the sulfate had not been completely deconjugated within the sewer network.

**Figure 5:**
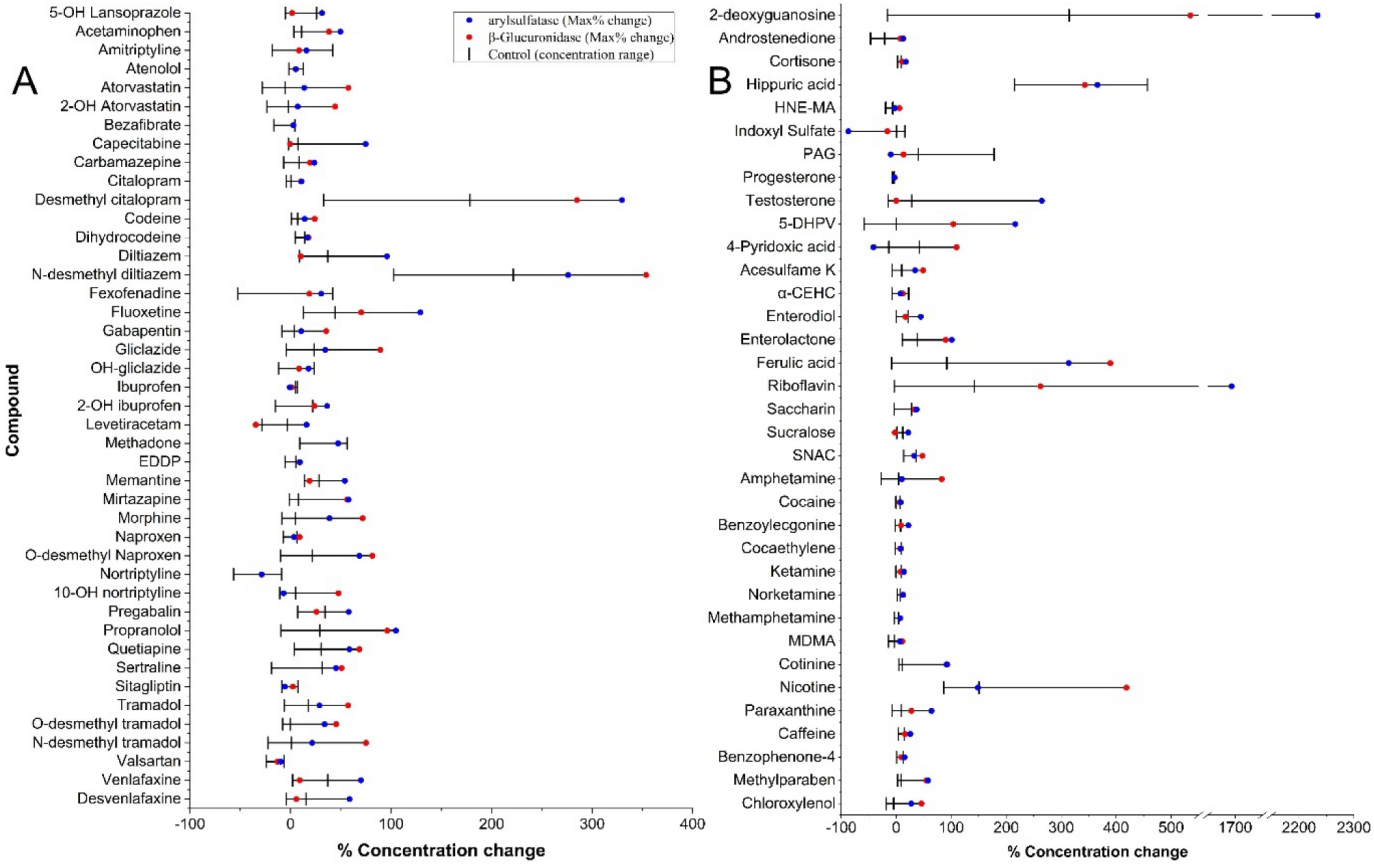
(A) Total observed deconjugation for all pharmaceuticals. (B) Total observed deconjugation for all other targets in this manuscript: illicit drugs, lifestyle chemicals, food, endogenous markers, and PCPs (where D,L-Sulforaphane-N-acetyl-l-cysteine = SAC, 5-(3′,4′-Dihydroxyphenyl)-γ-valerolactone = 5-DHPV, PAG = phenylacetylglutamine).

The results in Figure 6 indicate the following trend of phase II metabolite persistence in wastewater: acyl-glucuronide < O-glucuronide < O-sulfate < N-glucuronide. Figure 6 reinforces that there is no measured concentration increase in wastewater for acyl-glucuronides (*n* = 3, bezafibrate, ibuprofen, and naproxen). This is followed by O-glucuronides (*n* = 10, 10-OH nortriptyline, 2-OH ibuprofen, atenolol, codeine, desvenlafaxine, dihydrocodeine, morphine, O-desmethyl tramadol, propranolol, and sucralose), where the mean (experimental concentration increase in wastewater/theoretical concentration increase in urine (%)) was 9.2%, and a large range was observed, indicating the importance of considering the rest of the analyte’s structure prior to assuming complete deconjugation. For example, morphine glucuronide exhibits resistance to complete in-sewer deconjugation, while acetaminophen glucuronide, a less sterically hindered molecule, is highly unstable in wastewater.^66^ O-glucuronide was followed by O-sulfate (*n* = 5, acetaminophen, cortisone, ferulic acid, O-desmethyl naproxen, and testosterone), where the mean was 42.9%. Here a smaller range was observed due to lower sulfate enzymatic activity within the sewer network. For acetaminophen, complete deconjugation of acetaminophen O-glucuronide was assumed due to previous literature reports.^66^ Lastly, the most persistent was N-glucuronide (*n* = 6, carbamazepine, citalopram, cotinine, fluoxetine, memantine, and nicotine), where the mean was 44.2%. Again, there is a chance for variability; however, N-glucuronides appear to show increased persistence within the sewer network. In all scenarios, it is expected that the free analyte will have a significant impact on conjugate stability. Due to no statistical significance observed in Figure 6, we acknowledge that increasing the current sample size (*n* = 24), with analytes that have a broad range of chemical structures alongside further mechanistic work, is required to fully elucidate the relationship between the conjugates. It is possible that when the sample size is increased, the established relationship may not hold (acyl-glucuronide < O-glucuronide < O-sulfate < N-glucuronide); however, despite this possibility, the work clearly demonstrates the importance of greater understanding of enzymatic deconjugation in wastewater. It is also important to note the small number of participants in urinary studies, and thus the excretion values may not be representative of community-level excretion as observed in the analysis of community wastewater.

**Figure 6:**
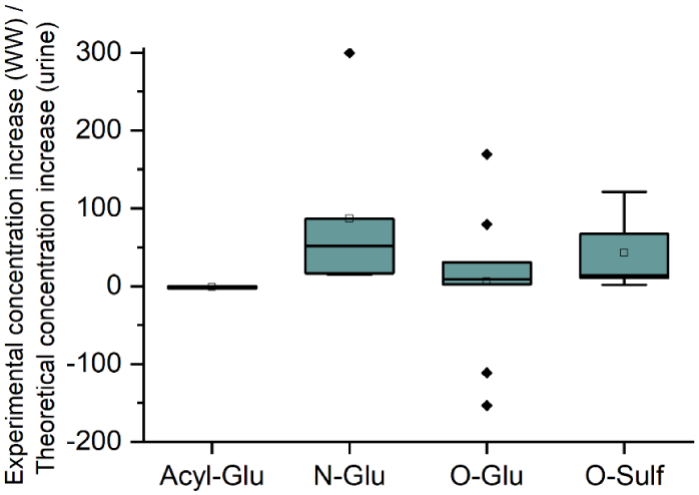
Comparison of the concentration increase observed in wastewater (WW) and urine following enzymatic deconjugation for Acyl-, O-, and N-glucuronides and O-sulfates.

The enzymatic deconjugation results were combined with the stability of free analytes in wastewater to identify compounds that may be problematic for routine analysis and could require further sample preparation via enzymatic deconjugation. Maximum concentration change following deconjugation (Table S4) was plotted against maximum change in concentration during the stability study (Table S6), as described in Section 2.4. Similar to stability, where McCall et al. defined three main categories of stability: low (60–100% transformation), medium (20–60% transformation), and high stability (0–20% transformation).^55^ Here, a framework with the same bands is used to identify the accuracy of predicting the concentration of the free analyte with conventional analytical methods. Data from Figure 7 and Table S5 were combined to provide a comprehensive recommendation of which analytes are suitable to analyze with current methods and those free analytes that may be at risk of over- or underprediction.

**Figure 7:**
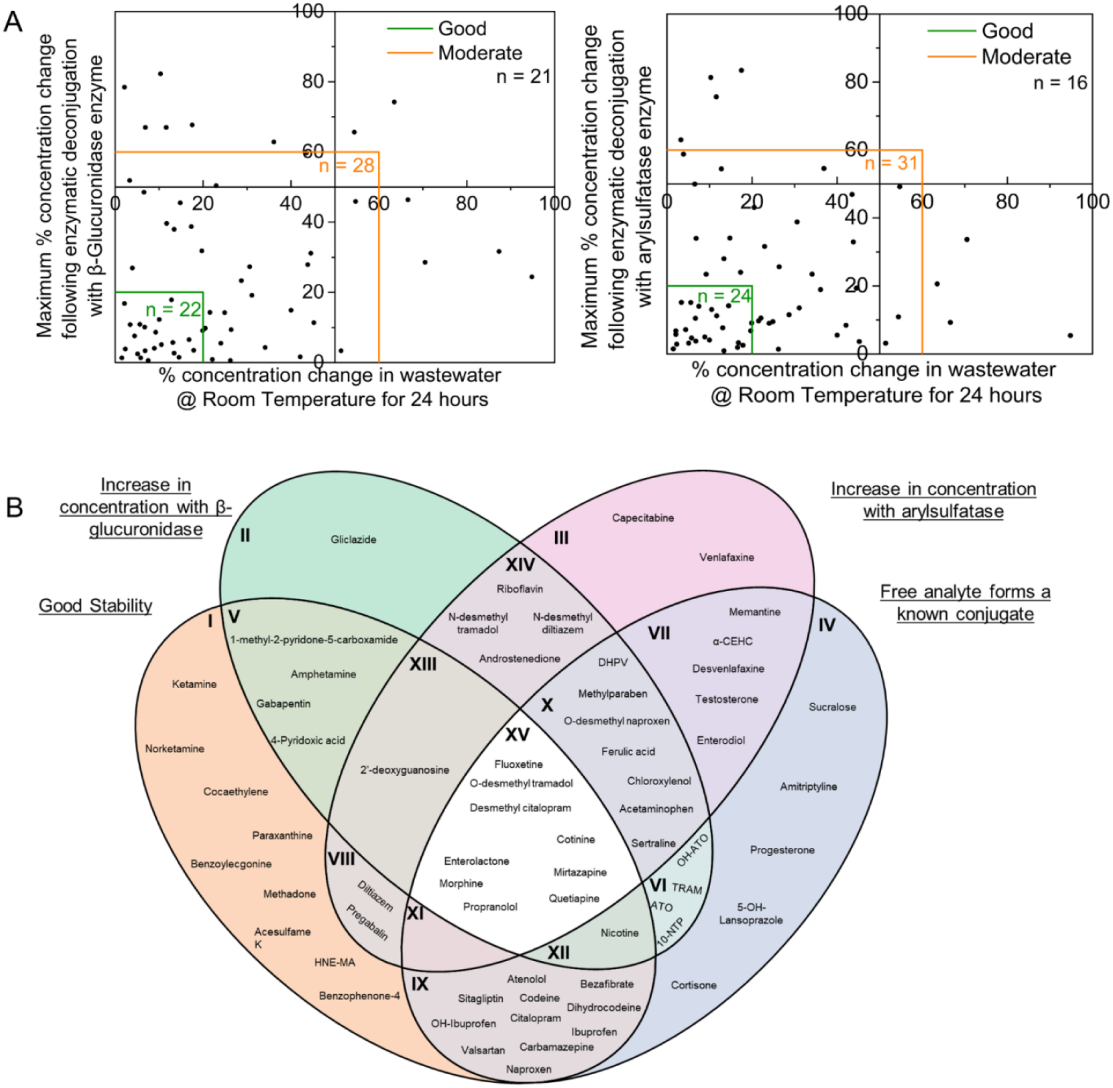
(A) Comparison between the potential impact of stability and enzymatic deconjugation on the quantification of analytes within WBE. Left = β-glucuronidase and right = arylsulfatase enzymes. (B) Summary of Sections 3.1–3.4 where the Venn diagram demonstrates the potential impact that stability and treating wastewater samples with enzymes have on phase II metabolites and other free analytes. ATO = Atorvastatin, OH-ATO = 2-hydroxy atorvastatin, TRAM = tramadol, and 10-NTP = 10-hydroxy nortriptyline. A description of the subgroups can be found in Table S7. Figures S20–S23 highlight the individual subgroups.

Figure 7A in tandem with Tables S4 and S6 demonstrates that 12/36 compounds with good stability were at risk of variability following enzymatic deconjugation. Additionally, three compounds—codeine, citalopram, and carbamazepine—all have known conjugates and exhibited >10% change in concentration. It is possible that in certain sewer networks, with reduced hydraulic retention times, these compounds are at risk of underestimation. Figure 7B acts as a complete summary of this work, identifying how these targets fit into four main categories: good stability, a known conjugate, and demonstrated an increase in concentration with either enzyme under study. Of the 79 analytes, 12 did not fit into any category. This includes compounds that are already phase II metabolites, such as indoxyl sulfate, hippuric acid, and phenylacetyl glutamine. Others include saccharin, nortriptyline, OH-gliclazide, fexofenadine, caffeine, MDMA, methamphetamine, and cocaine. The variable concentration after deconjugation was also accounted for. The central zone, section XV, contains targets that have known conjugates, have good stability, and increase in concentration with both enzymes. As these are targets, which are routinely analyzed in WBE, additional sample preparation steps, such as enzymatic deconjugation or a separate additional study into the phase II metabolites themselves, should be considered. There were also multiple instances where an analyte had no known conjugate; however, a change in concentration was observed. This could indicate possible transformations occurring at higher enzyme concentrations—these certain analytes need to be treated with caution; however, it is possible that these reactions could be due to side reactions of the enzymes. Here, these analytes would not be able to be deployed into the same sample preparation framework as those that require enzymatic deconjugation. Increased deconjugation could occur in warmer climates, and therefore certain analytes, displayed in sections II, III, V, VIII, XIII, and XIV in Figure 7B may have increased variability. Certain analytes with known conjugates (IV, VI, VII, IX–XII, and XV in Figure 7B) were expected to increase in concentration following enzymatic deconjugation; this was independent of the free analyte stability, where VI, VII, and X had a total of 16 analytes.

Overall, Sections 3.1–3.4 demonstrated the importance of understanding phase II metabolism within the context of sample preparation and the possible over- or underestimations that could occur if researchers assume complete deconjugation for certain analytes, as outlined in Figures 4–7. A direct comparison of both bulk stability and transformation to signpost the suitability for future analysis is provided below in Table 3.

**Table 3:**
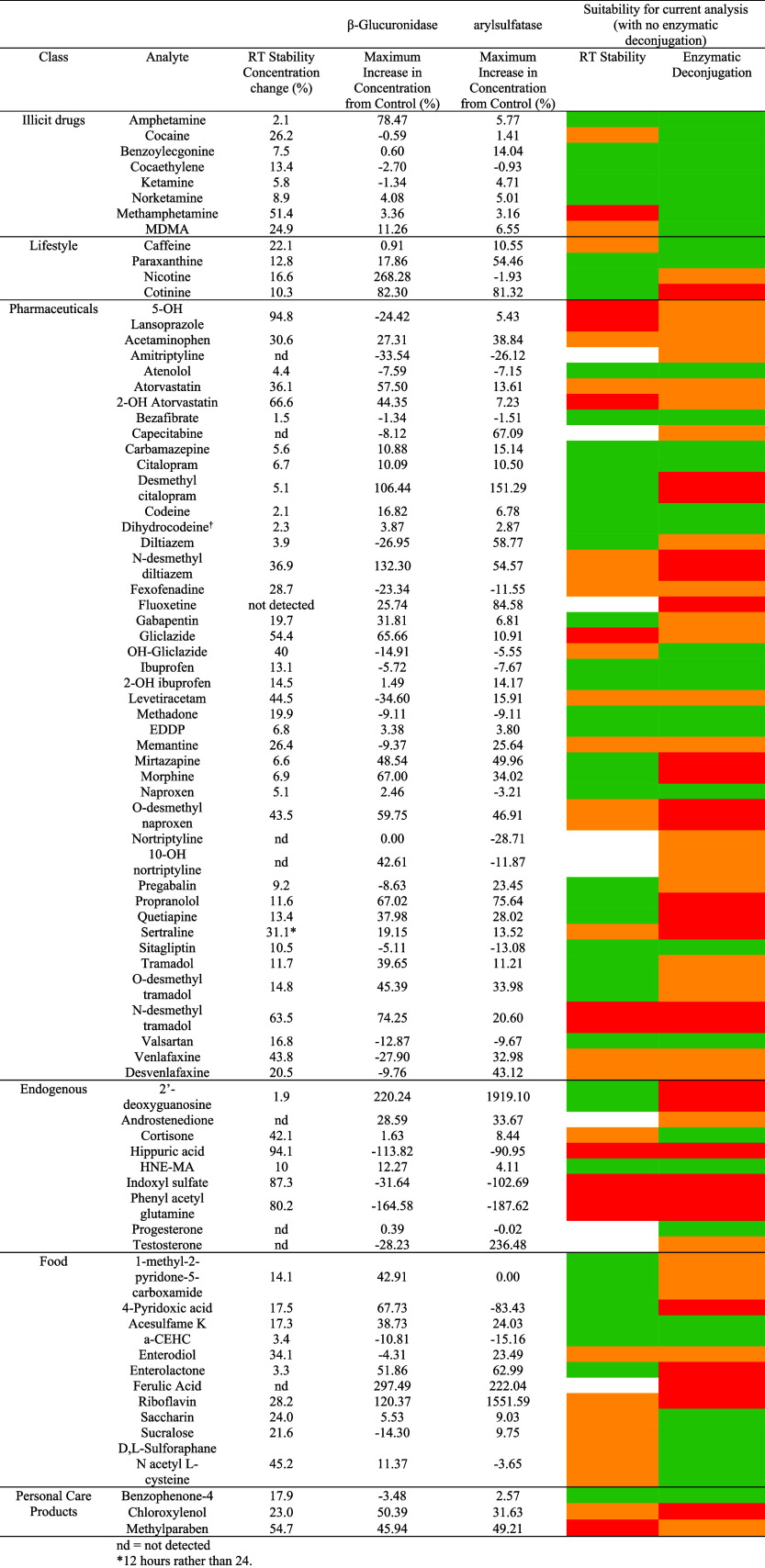
Direct Comparison between Variability Due to Stability and Enzymatic Deconjugation^a^

a Suitability columns are colored to indicate if the analyte can be analyzed via current methods (green = yes, orange = variability, proceed with caution, red = no). The source of the stability study is found in Table S6.

Due to the variability exhibited in Figures S6–S17, coupled with no plateau reached as indicated in Table S5, some analytes were reclassified due to a lack of certainty around their suitability. Future work will identify a new, robust metric to account for variability. Table 3 clearly demonstrates which analytes are at risk of underestimation in both WBE and ERA, and these chemicals require further investigation.

### 3.6 In-Freezer Stability of Phase II Metabolites

Another important aspect that must be considered is the stability of these analytes upon storage. Samples are often frozen prior to analysis, either to transport to the analyzing laboratory or for creating a sample repository. While the stability of free analytes upon storage has been studied before, the stability of phase II metabolites is often overlooked.^9^

Free and conjugated stability was assessed to see if phase II metabolites degraded upon storage, increasing the concentration of free analyte and limiting the potential analysis of phase II metabolites in the future. Figure 8 and Figures S24–S26 show that, for the analytes under study, the stability of the phase II metabolite in the freezer appears to be independent of the conjugate type. The stability of the conjugates was generally found to be dependent on the stability of the free analyte itself, indicating that at lower temperatures (−28 °C), the enzymatic activity has been reduced.

**Figure 8:**
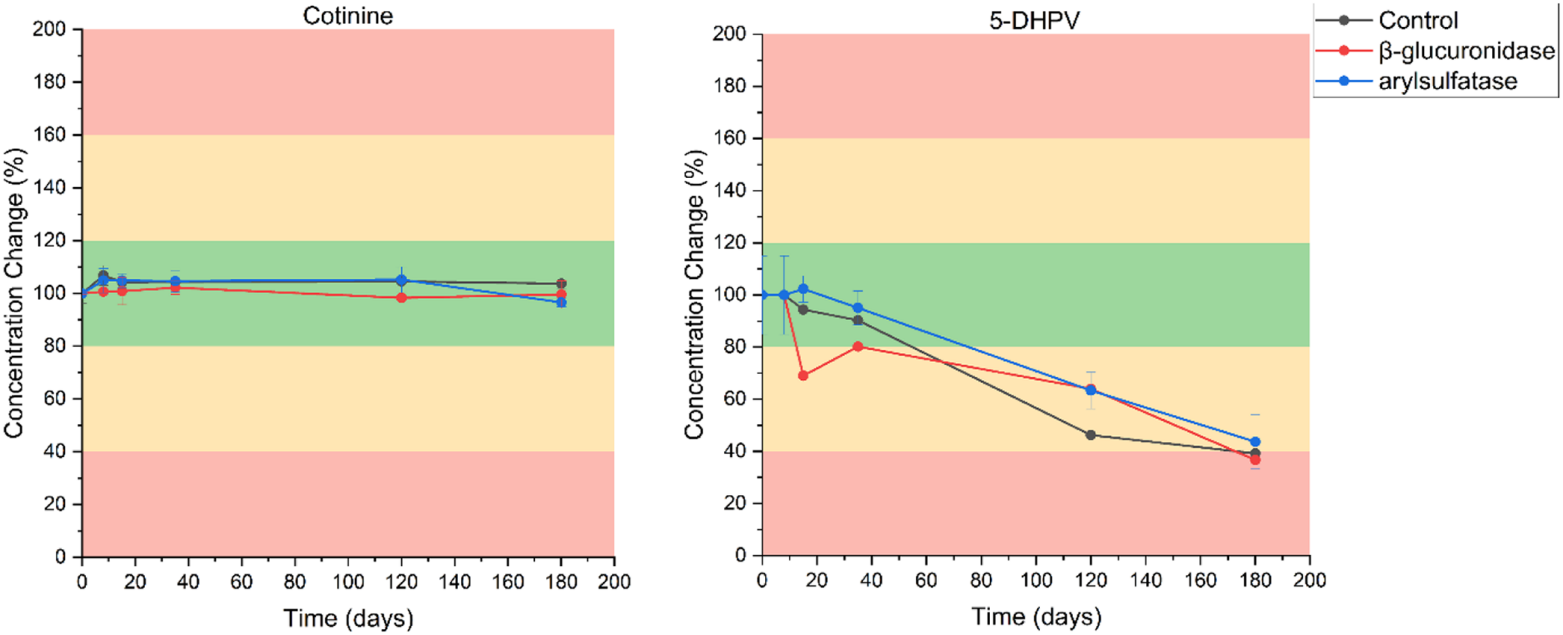
Concentration–time plot of the in-freezer stability of cotinine (left) and 5-DHPV (right) and their phase II metabolites.

Cotinine is well-known to be stable, and the deconjugation of its phase II metabolite also does not vary the concentration when compared to T_0_ (noted by the lack of change in cotinine concentration). On the other hand, 5-DHPV, a marker for epicatechin consumption, has poor stability as both a free analyte and following deconjugation. The same rate of in-freezer degradation between the control and deconjugated samples reinforces that in-freezer stability is dependent on the structure of the target itself rather than the type of conjugation. While this trend was noted for the comprehensive selection of WBE targets included in this work, future studies should evaluate the veracity of this trend with additional markers and phase II metabolites.

### 3.7 Limitations

A wider limitation of the analysis of both phase I and phase II metabolites in WBE is the variability in adsorption, distribution, metabolism, and excretion of people within different communities, as it is likely not represented in individual excretion studies, as they often contain a low number of participants (typically *n* < 10). To address this, WBE researchers have attempted to determine new, more representative correction factors (incorporating excretion rate and stability, among others) by comparing prescription or sales data to the wastewater load.^33,71^ However, these approaches present their own limitations, assuming complete adherence to prescriptions, whereas 50% of prescriptions are not taken as prescribed.^72^ Additionally, there are many factors that have wider implications on excretion rates, which can act as limitations in WBE analysis.

The type of phase II conjugate formed is dependent on gender and genetics.^73,74^ A study conducted monitoring bisphenol A exposure, via their phase II metabolites, highlighted that there were significant differences in the conjugate formed.^75^ Here, males excreted bisphenol A as glucuronide conjugates, whereas females excreted it, primarily, as sulfate conjugates.^75^ Additional work studying acetaminophen indicated a significant increase in glucuronide conjugation in males; however, there was no change in sulfonation.^76^ Increased glucuronidation in males has also been observed in other studies, both in humans^77^ and in rats.^78^ There are often significant changes in metabolism following the consumption of other pharmaceuticals or plant-derived natural products.^76,79–81^ This is particularly relevant as approximately a quarter of the UK population takes at least three prescriptions.^82^

Studies have suggested that there are links between diet and the extent of phase II metabolism via transferase enzymes.^83–85^ This would require further work to understand because this could lead to significant variability in chemical metabolism at the catchment level, thereby directly affecting WBE analyses. Here, the socio-economic classes of a given community can have an impact on the consumption of fresh foods (fruits and vegetables) and, as a result, impact metabolism rates. Alongside dietary factors affecting the function of glucuronosyltransferase enzymes, there is also an impact on the concentrations of β-glucuronidase in feces.^86^ Therefore, increased concentrations may result in increased in-sewer deconjugation. These factors may limit the impact of the quantification of community-level pharmaceutical consumption and chemical exposure and, therefore, require further study.

Alongside community-level changes in metabolism, this article assessed stability only within bulk wastewater liquid. The impact of rising main or gravity sewers and the impact of biofilms should also be explored in future work. In-lab stability studies have demonstrated that these factors can impact the stability of analytes and, therefore, may also affect phase II metabolites.^87^ This work demonstrates the impact of temperatures on enzyme activity at 37 °C compared to in-freezer temperatures (−28 °C); however, temperatures between these values were not assessed. Due to the variable temperatures expected within sewer networks, the rate of deconjugation may be decreased in colder climates; however, it is not known at which temperature this activity in the sewer network will be significantly reduced.

It is important to note that the conclusions of this article are dependent on the analytes under study, and it is possible that other analytes may follow different trends. Commonly analyzed targets were used here to ensure the broad applicability of these results and to highlight the impact of this work. Additional research is required to investigate additional compounds. The conclusions obtained here are also reflective of this sewer network under study; it is possible that other networks with variable characteristics (including, but not limited to, hydraulic retention time, biofilm, and wastewater flow rate) may lead to different conclusions. Future experiments could use sampling across points of a sewer network^88^ to detect in situ deconjugation, which would facilitate a greater understanding of deconjugation.

### 3.8 Impact of Chemical Metabolism on WBE and ERA

This work provides a deeper understanding of the potential impact of phase II metabolism on the analysis of targets within an environmental or WBE context. This investigation examined two major phase II metabolites, glucuronides and sulfates, including multiple binding sites (acyl, O-, and N-glucuronides), to highlight the need for careful consideration before assuming complete in-sewer deconjugation. This work demonstrates that in-sewer stability for glucuronide conjugates varies from acyl < O < N, with sulfates also demonstrating strong persistence. The inclusion of common WBE targets, which observed no change during deconjugation, highlights that enzymatic deconjugation can be incorporated into workflows without hindering the quantification of other analytes. Additionally, the stability of these conjugates was investigated upon freezing for sample storage, where reduced enzymatic activity causes any instability to be attributed to the parent analyte itself rather than the conjugate. During WBE and ERA, it is critical to account for phase II metabolites, as it could lead to an underestimation of a chemical, leading to errors in public health and environmental risk assessments.

## Supplementary Material



## Notes

Details of all analytes under study, including class, analyte description, analyte type, structural formula, CAS-No, and supplier (Table S1); literature search of type of phase II metabolite, analyte excretion, and phase II metabolite excretion for all analytes under study (Table S2); literature search of other wastewater and urinary studies which have performed enzymatic deconjugation for a wide range of compounds (Table S3); structures of chemical targets, including location of conjugation for targets which form acyl-glucuronide conjugates (Figure S1); structures of chemical targets, including location of conjugation for targets which form N-glucuronide conjugates, includes proposed location of possible conjugation when not reported in literature (Figure S2); structures of chemical targets, including location of conjugation for targets which form O-glucuronide conjugates, includes proposed location of possible conjugation when not reported in literature (Figure S3); structures of chemical targets, including location of conjugation for targets which form O-sulfate conjugates, includes proposed location of possible conjugation when not reported in literature (Figure S4); structures of chemical targets, including location of conjugation for targets which form N-carbamoyl conjugates (Figure S5); results table for increases in concentration over time for control and enzyme experiments (Table S4); time–concentration plots for all analytes following enzymatic deconjugation (Figures S6–S18); statistical test to determine if the concentration had plateaued over time following deconjugation (Table S5); LC-MS/MS traces of 8-iso-PGF_2β_ after enzyme treatment (Figure S19); stability of analytes under study in bulk wastewater (Table S6); classification of subclasses from Venn diagram in Figure 7B (Table S7); individual breakdown of each subsection from Venn diagram in Figure 7B (Figures S20–S23); time–concentration plots for analytes during the in-freezer stability study (Figures S24–S26) (PDF)
