# Supplementary material for: Understanding the Role of Deconjugation of Phase II Metabolites in Wastewater: Implications for Wastewater-Based Epidemiology
Source: Environ Sci Technol. 2026 Feb 9;60(7):5789–803. doi: 10.1021/acs.est.5c17466 (PMC12947683; doi:10.1021/acs.est.5c17466)
Supplement: Supplementary file 1 [file es5c17466_si_001.pdf]

Understanding the role of deconjugation of phase-II metabolites in wastewater: implications to Wastewater-Based Epidemiology

Harry Elliss[a,b,c], Katarina Hricova[a], Evie Griffiths[a], Neil Andrew Byrnes[a,b,c,d], Ben Faill[a,b,c], Eva Hawkins[a,b,c], Kit Proctor[e], Megan Robertson[b,f], John Bagnall[b,f], Barbara Kasprzyk-Hordern[a,b,c]*

[a] *Department of Chemistry, University of Bath, Claverton Down, Bath, BA2 7AY, UK*

[b] *Centre of Excellence in Water-Based Early-Warning Systems for Health Protection, University of Bath, Claverton Down, Bath, BA2 7AY, UK*

[c] *Institute of Sustainability and Climate Change, University of Bath, Claverton Down, Bath, BA2 7AY, UK*

[d] *Australian Centre for Research on Separation Science, School of Chemistry, Monash University, Wellington Road, Clayton, Victoria 3800, Australia*

[e] *Chemical Characterisation Facility, University of Bath, Claverton Down, Bath, BA2 7AY, UK*

[f] *Wessex Water Service Ltd., Claverton Down, Bath, BA2 7WW, UK*

*Author for correspondence

E-mail address: bkh20@bath.ac.uk (Barbara Kasprzyk-Hordern)

Table S11. Analyte list and description for biomarkers under study within this manuscript, suppliers for all targets and internal standards

Analyte	Class	Analyte description	Analyte type	Structural Formula	CAS-No	Supplier
Amphetamine	Illicit drug	stimulant	parent	C9H13N	300-62-9	LGC (cerilliant)
Cocaine		stimulant	parent	C17H21NO4	50-36-2	LGC (cerilliant)
Benzoylecgonine		stimulant	metabolite	C16H19NO4	519-09-5	Sigma Aldrich (cerilliant)
Cocaethylene		stimulant	metabolite	C18H23NO4	529-38-4	Sigma Aldrich (cerilliant)
Ketamine		stimulant	parent	C13H16ClNO	6740-88-1	Sigma Aldrich
Norketamine		stimulant	metabolite	C12H14ClNO	35211-10-0	Sigma Aldrich
Methamphetamine		stimulant	parent	C10H15N	537-46-2	LGC (cerilliant)
MDMA		stimulant	parent	C11H15NO2	42542-10-9	LGC
Caffeine	Lifestyle	-	parent	C8H10N4O2	58-08-2	Sigma Aldrich
Paraxanthine		caffeine consumption	metabolite	C7H8N4O2	611-59-6	Sigma Aldrich
Nicotine		-	parent	C10H14N2	54-11-5	Sigma Aldrich (cerilliant)
Cotinine		nicotine consumption	metabolite	C10H12N2O	486-56-6	Sigma Aldrich
5-OH Lansoprazole	Pharmaceutical	proton pump inhibitor	metabolite	C16H14F3N3O3S	131926-98-2	LGC
Acetaminophen		analgesics	parent	C8H9NO2	103-90-2	Sigma Aldrich
Amitriptyline		anti-depressant	parent	C20H23N	50-48-6	Sigma Aldrich
Atenolol		Beta blocker	parent	C14H22N2O3	29122-68-7	Sigma Aldrich
Atorvastatin		anti-hyperlipidemic	parent	C33H35FN2O5	134523-00-5	LGC
2-OH Atorvastatin		anti-hyperlipidemic	metabolite	C33H35FN2O6	265989-46-6	Sigma Aldrich
Bezafibrate		antihyperlipidemic	parent	C19H20ClNO4	41859-67-0	Sigma Aldrich
Capecitabine		Chemotherapy	parent	C15H22FN3O6	154361-50-9	Sigma Aldrich
Carbamazepine		anti-convulsant	parent	C15H12N2O	298-46-4	Sigma Aldrich
Citalopram		anti-depressant	parent	C20H21FN2O	59729-33-8	Sigma Aldrich
Desmethyl citalopram		anti-depressant	metabolite	C19H19FN2O	62498-67-3	TRC
Codeine		opioid	parent	C18H21NO3	76-57-3	Sigma Aldrich
Dihydrocodeine		opioid	parent & metabolite	C18H23NO3	125-28-0	Sigma Aldrich
Diltiazem		Calcium channel blocker	parent	C22H26N2O4S	42399-41-7	Sigma Aldrich
N-desmethyl diltiazem		Calcium channel blocker	metabolite	C21H24N2O4S	86408-45-9	Sigma Aldrich
Fexofenadine		anti-histamine	parent	C32H39NO4	83799-24-0	LGC
Fluoxetine		anti-depressant	parent	C17H18F3NO	54910-89-3	Sigma Aldrich
Gabapentin		anti-convulsant	parent	C9H17NO2	60142-96-3	Sigma Aldrich
Gliclazide		anti-hyperglycemic	parent	C15H21N3O3S	21187-98-4	LGC
OH-Gliclazide		anti-hyperglycemic	metabolite	C15H21N3O4S	87368-00-1	Sigma Aldrich
Ibuprofen‡		non-steroidal anti-inflammatory drug	parent	C13H18O2	15687-27-1	Sigma Aldrich
2-OH ibuprofen‡		non-steroidal anti-inflammatory drug	metabolite	C13H18O3	51146-55-5	Sigma Aldrich
Levetiracetam		anti-convulsant	parent	C8H14N2O2	102767-28-2	Sigma Aldrich
Methadone		opiod	parent	C21H27NO	76-99-3	Sigma Aldrich (cerilliant)
EDDP		opiod	metabolite	C21H27NO	30223-73-5	Sigma Aldrich (cerilliant)
Memantine		NMDA receptor antagonist	parent	C12H21N	19982-08-2	Sigma Aldrich
Mirtazapine		anti-depressant	parent	C17H19N3	85650-52-8	Sigma Aldrich
Morphine		opioid	parent	C17H19NO3	57-27-2	Sigma Aldrich
Naproxen		non-steroidal anti-inflammatory drug	parent	C14H14O3	22204-53-1	TRC
O-desmethyl naproxen		non-steroidal anti-inflammatory drug	metabolite	C13H12O3	52079-10-4	Sigma Aldrich (cerilliant)
Nortriptyline		anti-depressant	parent	C19H21N	72-69-5	Sigma Aldrich
10-OH nortriptyline		anti-depressant	metabolite	C19H21NO	47132-16-1	TRC
Pregabalin		anti-convulsant	parent	C8H17NO2	148553-50-8	Sigma Aldrich (cerilliant)
Propranolol		Beta blocker	parent	C16H21NO2	526-66-6	Sigma Aldrich
Quetiapine		anti-psychotic	parent	C21H25N3O2S	111974-69-7	LGC
Sertraline		anti-depressant	parent	C17H17Cl2N	79617-96-2	Sigma Aldrich
Sitagliptin		anti-hyperglycemic	parent	C16H15F6N5O	486460-32-6	TRC
Tramadol		opiod	parent	C16H25NO2	27203-92-5	Sigma Aldrich
O-desmethyl tramadol		opiod	metabolite	C15H23NO2	80456-81-1	LGC
N-desmethyl tramadol		opiod	metabolite	C15H23NO2	73806-55-0	LGC
Valsartan		anti-hypertensive	parent	C24H29N5O3	137862-53-4	Sigma Aldrich
Venlafaxine		anti-depressant	parent	C17H27NO2	93413-69-5	Sigma Aldrich
Desvenlafaxine		anti-depressant	metabolite	C16H25NO2	93413-62-8	Sigma Aldrich
2'-deoxyguanosine	Human markers	oxidative stress	metabolite	C10H13N5O4	961-07-9	Sigma Aldrich
Androstenedione		hormone	metabolite	C19H26O2	63-05-8	Sigma Aldrich
Cortisone		hormone	metabolite	C21H28O5	53-06-5	Sigma Aldrich

Analyte	Class	Analyte description	Analyte type	Structural Formula	CAS-No	Supplier
Hippuric acid		biomarker of metabolic health	metabolite	C9H9NO3	495-69-2	Sigma Aldrich
HNE-MA		biomarker of lipid peroxidation	metabolite	C14H25NO5S	146764-24-1	Cayman chemical
Indoxyl sulfate		metabolite of dietary tryptophan	metabolite	C8H7NO4S	487-94-5	Cayman chemical
Phenyl acetyl glutamine		human metabolite	metabolite	C13H16N2O4	28047-15-6	TRC
Progesterone		hormone	metabolite	C21H30O2	57-83-0	Sigma Aldrich
Testosterone		hormone	metabolite	C19H28O2	58-22-0	Sigma Aldrich
1-methyl-2-pyridone-5-carboxamide	Food	metabolite of nicotinamide	metabolite	C7H8N2O2	701-44-0	TRC
4-Pyridoxic acid		vitamin B6	metabolite	C8H9NO4	82-82-6	TRC
Acesulfame K		artificial sweetener	parent	C4H5NO4S	33665-90-6	Cayman chemical
a-CEHC		metabolite of vitamin E	metabolite	C16H22O4	4072-32-6	Cayman chemical
Enterodiol		polyphenol metabolite	metabolite	C18H22O4	80226-00-2	Sigma Aldrich
Enterolactone		polyphenol metabolite	metabolite	C18H18O4	78473-71-9	Sigma Aldrich
Ferulic Acid		phenolic acid found in multiple foods	parent	C10H10O4	1135-24-6	Sigma Aldrich
Riboflavin		vitamin B2	metabolite	C17H20N4O6	83-88-5	TRC
Saccharin		artificial sweetener	parent	C7H5NO3S	81-07-2	Sigma Aldrich
Sucralose		artificial sweetener	parent	C12H19Cl3O8	56038-13-2	TRC
D,L-Sulforaphane N acetyl L-cysteine		food	metabolite	C11H20N2O4S3	334829-66-2	TRC
Benzophenone-4	Personal care Product	UV filter	parent	C14H12O4S	4065-45-6	Sigma Aldrich
Chloroxylenol		anti-septic	parent	C8H9ClO	88-04-0	Sigma Aldrich
Methylparaben		paraben	parent	C8H8O3	99-76-3	Sigma Aldrich
Acetaminophen-d4	ISTD		parent	C8D4H5NO2	64315-36-2	Sigma Aldrich
Amitriptyline-d3 (HCl)			parent	C20D3H20N	342611-00-1	Sigma Aldrich
Amphetamine-d5			parent	C9H8D5N	65538-33-2	Sigma Aldrich
Atenolol-d7			parent	C14H15D7N2O3	1202864-50-3	Sigma Aldrich
Benzoylecgonine-d8			metabolite	C16H11D8NO4	205446-21-5	Sigma Aldrich
Bezafibrate-d6			parent	C19H14D6ClNO4	1219802-74-0	QMX laboratories
Bisphenol A-d16			parent	C15 2H16 O2	96210-87-6	Sigma Aldrich
Caffeine-d9			parent	C8H1D9N4O2	72238-85-8	Sigma Aldrich
Carbamazepine-13C6			parent	13C6C9H12N2O	-	Sigma Aldrich
Citalopram-d6			parent	C20H15D6FN2O	1190003-26-9	Sigma Aldrich
Cocaethylene-d3			metabolite	C18D3H20NO4	136765-30-5	Sigma Aldrich
Cocaine-d3			parent	C17D3H18NO4	65266-73-1	Sigma Aldrich
Codeine-d6			parent	C18H21NO3	1007844-34-9	Sigma Aldrich
Cortisol-d4			parent	C21H26D4O5	73565-87-4	Sigma Aldrich
Cotinine-d3			metabolite	C10H9D3N2O	110952-70-0	Sigma Aldrich
Desmethyl Diazepam-d5			metabolite	C15H6ClD5N2O	65891-80-7	Sigma Aldrich
Diazepam-d5			parent	C16H8D5ClN2O	65854-76-4	Sigma Aldrich
EDDP-d3			metabolite	C20H21ClNO4	136765-23-6	Sigma Aldrich
Estrone-d4			metabolite	C18D4H18O2	53866-34-5	Sigma Aldrich
Gabapentin-d4			parent	C9D4H13O2	1185039-20-6	TRC
Ibuprofen-d3			parent	C13D3H15O2	121662-14-4	Sigma Aldrich
Ketamine-d4 (HCl)			parent	C13D4H12ClNO	1867-66-9	Sigma Aldrich
MDMA-d5			parent	C11H10D5NO2	136765-43-0	Sigma Aldrich
Methadone-d9			parent	C21H18D9NO	1435933-74-6	Sigma Aldrich
Methamphetamine-d5			parent	C10H10D5N	60124-88-1	Sigma Aldrich
Methylparaben 13C6			parent	[13]C6C2H8O3	1581694-95-2	LGC
Metoprolol-d7			parent	C15D7H18NO3	959787-96-3	LGC
Mirtazapine-d3			parent	C17H16D3N	1216678-68-0	Sigma Aldrich
Morphine-d3			parent	C17H16D3NO3	67293-88-3	Sigma Aldrich
Naproxen-d3			parent	C14H11D3O3	958293-79-3	Sigma Aldrich
Norketamine-d4 (HCl)			metabolite	C13H10D4ClNO	1435934-57-8	Sigma Aldrich
Nortriptyline-d3 (HCl)			parent	C19H18D3N	203784-52-5	Sigma Aldrich
Propranolol-d7			parent	C16H14D7NO	98897-23-5	Sigma Aldrich
Quetiapine-d8 (hemifumerate)			parent	C21H17D8N3O2S	1185247-12-4	Sigma Aldrich
Sertraline-d3			parent	C17H14D3Cl2N	1217741-83-7	Sigma Aldrich

Table S2. Literature search of analytes under study in this manuscript and their conjugate type and excretion rate.

Analyte	Phase II Metabolite	Type of Phase II metabolite	Analyte excretion (%)	Phase II metabolite excretion (%)	Ref
Amphetamine	No[a]	-	36[b]	-	a) [1]<br>b) [2]
Cocaine	No[a, b]	-	7.5[c]	-	a) [3]<br>b) [4]<br>c) [5]
Benzoylecgonine	No[a, b]	-	29[c]	-	a) [3]<br>b) [4]<br>c) [6]
Cocaethylene	No	-	-	-	[7]
Ketamine	No[a]	-	30[b]	-	a) [8]<br>b) [9]
Norketamine	No[a]	-	1.6[b]	-	a) [8]<br>b) [10]
Methamphetamine	No[a]	-	41[b]	-	a) [11]<br>b) [2]
MDMA	No[a]	-	22.50[b]	-	a) [12]<br>b) [2]
Caffeine	No[a]	-	1.00[b]	-	a) [13]<br>b) [10]
Paraxanthine	No[a]	-	4.60[b]	-	a) [13]<br>b) [14]
Nicotine	Yes	N-glucuronide (quaternary)	8-10	3-5	[15]
Cotinine	Yes	N-glucuronide (quarternary)	10-15	12-17	[15]
5-OH Lansoprazole	Yes	Unavailable	15-23% as free + conjugate	-	[16]
Acetaminophen	Yes	1) O-glucuronide<br>2) Sulfate	3.55	1) 51.99<br>2) 27.34	[17]
Amitriptyline	Yes	N-glucuronide	0.08-1.68	8	[18]
Atenolol	Yes[a]	O-glucuronide[a]	41.60[b]	2[a]	a) [19]<br>b) [20]
Atorvastatin	Yes[a, b]	Acyl-glucuronide[a]<br>O-glucuronide[b]	2[c]	- [a]	a) [21]<br>b) [22]<br>c) [20]
2-OH Atorvastatin	Yes	Acyl-glucuronide<br>O-glucuronide	Unavailable	-	[22]
Bezafibrate	Yes[a]	Acyl-glucuronide[a]	44.13[b]	22.1[a]	a) [23]<br>b) [20]
Capecitabine	No[a]	-	2.93[b]	-	a) [24]<br>b) [25]
Carbamazepine	Yes	N-glucuronide	13.8	11	[26]
Citalopram	Yes	1) N-glucuronide<br>2) acyl-glucuronide	26	1) 12<br>2) 12	[27]
Desmethyl citalopram	Possible	Possible however, not quantified	19	-	[27]
Codeine	Yes	O-glucuronide	5-15	10-20	[28]
Dihydrocodeine[†]	Yes	O-glucuronide	31.1	27.7-31.5	[29]
Diltiazem	No[a, b]	-	3.42[c]	-	a) [30]<br>b) [31]<br>c) [20]
N-desmethyl diltiazem	No[a]	-	3.63[b]	-	a) [31]<br>b) [20]
Fexofenadine	No[a]	-	6.30[b]	-	a) [32]<br>b) [20]
Fluoxetine	Yes	N-glucuronide	11	7	[33]
Gabapentin	No[a]	-	77.17[b]	-	a) [34]<br>b) [17]
Gliclazide	No[a]	-	9.17[b]	-	a) [35]<br>b) [20]
OH-Gliclazide	No	-	16.19	-	[35]
Ibuprofen	Yes[a, b]	Acyl-glucuronide[a, b]	Negligible[c]	10-15[a, b]	a) [36]<br>b) [37]<br>c) [38]
2-OH ibuprofen	Yes	O-glucuronide	9	17	[39]
Levetiracetam	No	-	66	-	[40]
Methadone	No[a]	-	27.50[b]	-	a) [41]<br>b) [42]
EDDP	No[a]	-	55[b]	-	a) [41]<br>b) [42]

Analyte	Phase II Metabolite	Type of Phase II metabolite	Analyte excretion (%)	Phase II metabolite excretion (%)	Ref
Memantine	Yes	N-glucuronide	48	26	[43]
Mirtazapine	Yes	N-glucuronide (quarternary)	<1-4	1-21	[44]
Morphine	Yes	2 x O-glucuronides (3,6 position)	2.6	65 (3-O-glucuronide = 55, 6-O-glucuronide = 10)	[45]
Naproxen	Yes	Acyl-glucuronide<br>Iso-acyl-glucuronide	1-1.6	51.8-52.9<br>7.6-7.9	[46]
O-desmethyl naproxen	Yes	1) Acyl-glucuronide<br>2) Iso-acyl-glucuronide<br>3) Sulfate*<br>4) Acyl-glucuronide*<br>5) O-glucuronide*<br>6) Acyl-glucuronide-sulfate diconjugate*	0.6-1.1<br>10*	1) 12.6-17.7<br>2) 4.9-7.2<br>3) 50*<br>4) 10*<br>5) 6*<br>6) 4*	[47]
Nortriptyline	No	-	-	-	[48]
10-OH nortriptyline	Yes[a]	O-glucuronide[a]	2-10[a, b]	1-5[a]	a) [49]<br>b) [50]
Pregabalin	No[a]	-	71.70[b]		a) [51]<br>b) [20]
Propranolol	Yes	O-glucuronide	8.3-15	4.33-16.58	[20]
Quetiapine	Yes	Unavailable	<1%	-	[52]
Sertraline	Yes[a]	Carbamoyl-O-glucuronide[a]	0.2%[b]	-	a) [53]<br>b) [54]
Sitagliptin	Yes[a]	N-sulfate[a]<br>N-carbamoyl glucuronide[a]	72.16[b]	<1-7[a]	a) [55]<br>b) [20]
Tramadol	Yes	1) Sulfate<br>2) Glucuronide (binding site not available)	25-30	1) <2<br>2) <2	[56]
O-desmethyl tramadol	Yes[a]	O-glucuronide[a]	14.95[b]	2-5[a]	a) [56]<br>b) [20]
N-desmethyl tramadol	No[a]	-	3.98[b]	-	a) [56]<br>b) [20]
Valsartan	Yes	Acyl-glucuronide<br>N-glucuronide	80%[††]	Low	[57]
Venlafaxine	No	-	4.7	-	[58]
Desvenlafaxine	Yes	O-glucuronide	29.4	26.4	[58]
2'-deoxyguanosine	-	-	-	-	
Androstenedione	No	-	-	-	[59]
Cortisone	Yes	Sulfate	-	300% increase in concentration observed	[60]
Hippuric acid	already conjugated (glycine)	-	-	-	-
HNE-MA	already conjugated (mercapturic acid)	-	-	-	-
Indoxyl sulfate	already conjugated (sulfate)	-	-	-	-
Phenyl acetyl glutamine	already conjugated (glutamine)	-	-	-	-
Progesterone	No	-	-	-	[59]
Testosterone	Yes	Sulfate	-	773% increase in concentration observed	[61]
4-Pyridoxic acid	-	-	-	-	-
Acesulfame K	No	-	-	-	[62]
a-CEHC	Yes	Taurine conjugate<br>Glycine conjugate<br>Glycine-glucuronide<br>Sulfate<br>Acyl glucuronide	-	-	[63]
Enterodiol	Yes	1) O-glucuronide<br>2) O,O-diglucuronide<br>3) Sulfate<br>4) Sulfoglucuronide<br>5) Disulfate	0.4	1) 81.1<br>2) 0.07<br>3) 6.8<br>4) 11.2<br>5) 0.45	[64]
Enterolactone	Yes	1) O-glucuronide<br>2) O,O-diglucuronide<br>3) Sulfate<br>4) Sulfoglucuronide<br>5) Disulfate	1.1	1) 91.6<br>2) 0.8<br>3) 3.0<br>4) 1.9<br>5) 1.6	[64]
Ferulic Acid	Yes	1) Glucuronide<br>2) Sulfate	26**	1) 17.7**<br>2) 55.6**	[65]
Riboflavin	-	-	-	-	-

Analyte	Phase II Metabolite	Type of Phase II metabolite	Analyte excretion (%)	Phase II metabolite excretion (%)	Ref
Saccharin	No	-	-	-	[66]
Sucralose	Yes	Glucuronide	98	2	[67]
D,L-Sulforaphane N acetyl L-cysteine	N-acetyl cysteine conjugate of sulforaphane	-	-	-	-
Benzophenone-4	-	-	-	-	-
Chloroxylenol	Yes‡	1) Glucuronide<br>2) Sulfate	0.1	1) 10.2<br>2) 43.3	[68]
Methylparaben	Yes	1) Glucuronide<br>2) Sulfate	-	-	[69]

† Dihydrocodeine dosed as parent compound
*O-desmethyl naproxen dosed as the parent compound
†† percentage calculated in bile
** combined concentrations of ferulic acid and isoferulic acid
‡ study conducted with dogs

Table S3. Other studies which performed enzymatic deconjugation with enzymes under different conditions for a range of compounds.

Matrix	Enzyme	Enzyme concentration / units per mL matrix	Incubation time	Temperature / °C	Compounds	Reference
Wastewater	β-Glucuronidase<br>α-Sulfatase		18 h	37	30 pharmaceuticals and 17 hormones	[70]
Wastewater (100 mL)	β-Glucuronidase from *Helix pomatia* type HP-2	200	30[(a)], 60, 90, 120 min	55	8-isoprostaglandin $F_2\alpha/\beta$ + benzodiazepines	[71]
Wastewater (100 mL)	β-Glucuronidase from *Helix pomatia* type H-2	425	1.5 h	37	Prostaglandin	[72]
Wastewater (100 mL)	β-Glucuronidase from *Helix pomatia* type H-2	425	2 h	37	8-isoprostaglandin	[73]
Wastewater (1.14 mL)	β-Glucuronidase from *Helix pomatia* type H-2	513	Between 2 and 5 h[(b)]	37	Nicotine and its metabolites	[74]
Urine (1 mL)	β-Glucuronidase from bovine liver type B-1, Sulfatase type V from *Helix pomatia*		3 h	37	Isoflavones	[75]
Urine (100 μL)	Solid β-glucuronidase from *Helix pomatia* type H-1, Sulfatase SH-1 from *Helix pomatia*	5000, 20000, 30000, 36000	4, 8, 24 or 48 h[(c)]	37	Parabens, triclocarban, phenols, personal care products	[76]
	Solid β-glucuronidase from *Helix pomatia* type H-1, liquid β-glucuronidase from E.coli BL-21	H-1: 5000 units/μL<br>BL-21: 0, 250, 500, 1000	4, 8, 24 or 48 h[(d)]	37		
	β-Glucuronidase from *Helix pomatia* type HP-2	2000, 4000, 6000	3.5	37		
Urine (300 μL)	β-Glucuronidase from *Helix pomatia* type HP-2	2 000	3.5 h	37	Paracetamol/ acetaminophen	[77]
Urine (0.2 mL)	recombinant β-glucuronidase from E.coli type IX-A	10 000	16 h	37	Cannabinoids	[78]

(a) preferred
(b) optimised at 5 h
(c) complete deconjugation at 30 units/ μL with 4 h
(d) complete deconjugation at 48 h

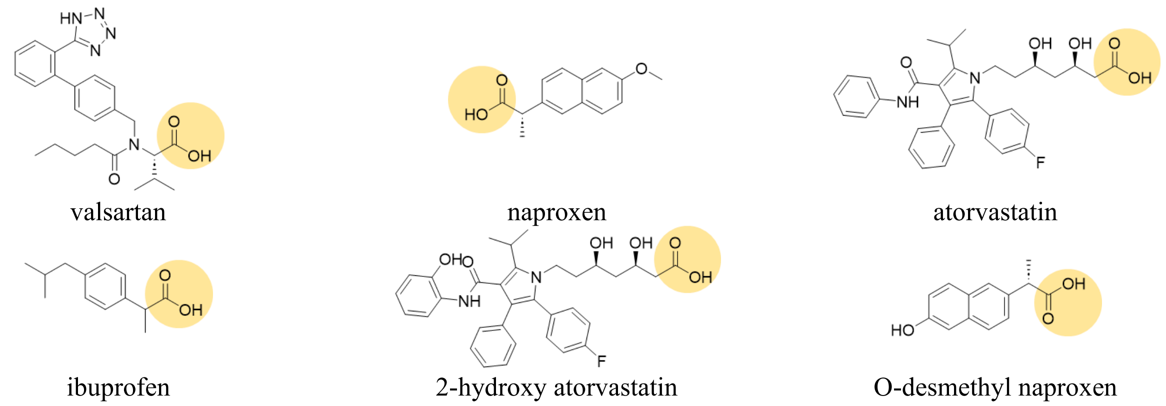

Figure S1. Chemical targets under study that form acyl-glucuronide conjugates. The highlighted functional group indicates the location of conjugation.

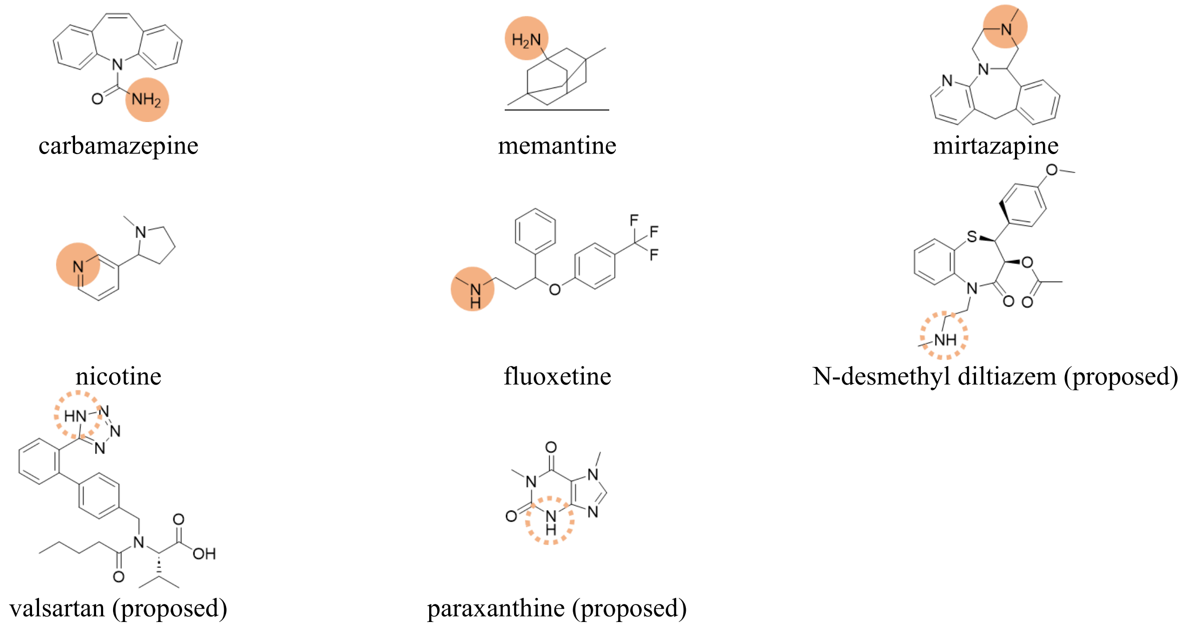

Figure S2. Chemical targets under study that form N-glucuronide conjugates. The highlighted functional group indicates the location of conjugation. Dashed circles demonstrate the possible location of conjugation however, the exact, known, location is not reported.

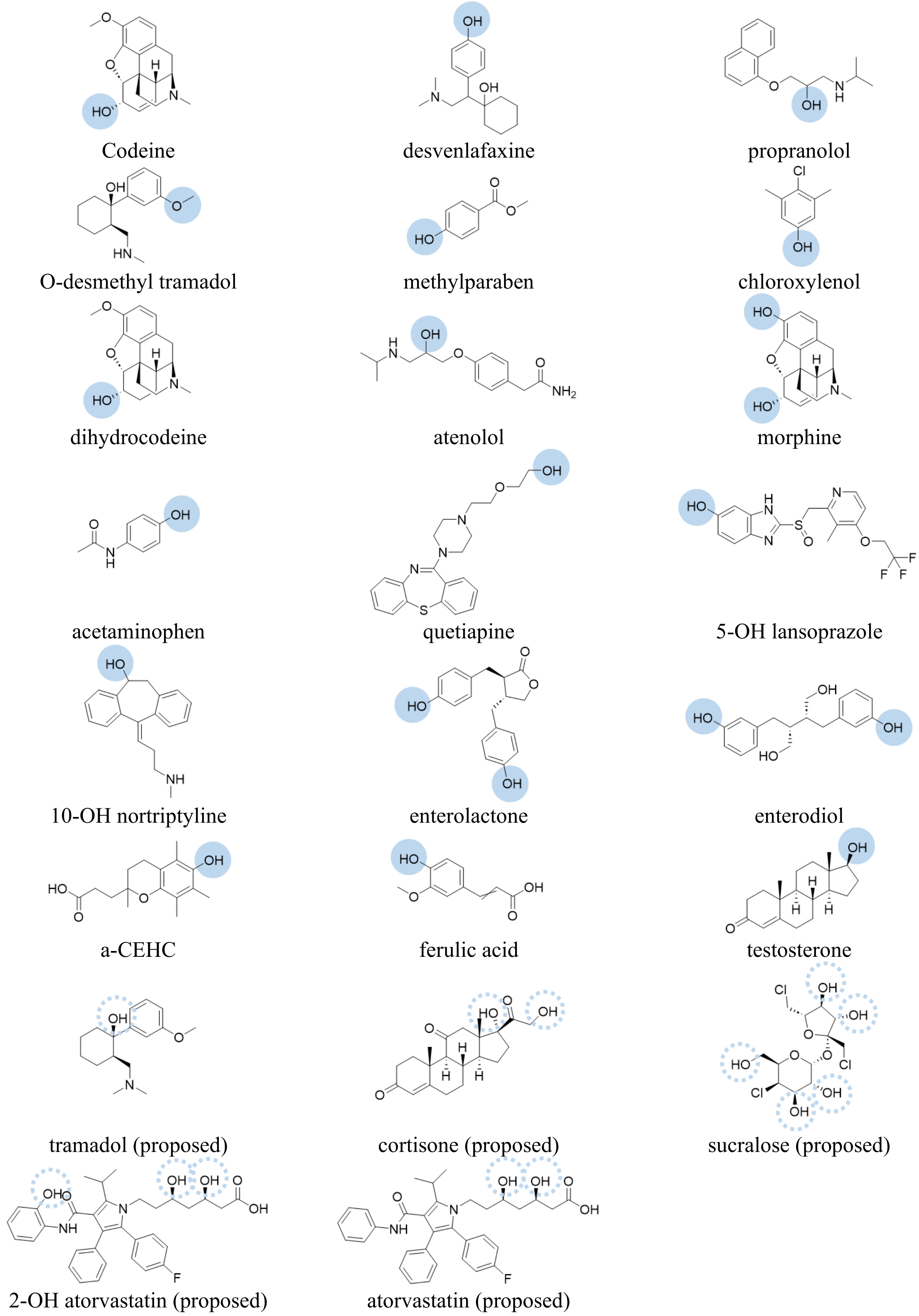

Figure S3. Chemical targets under study that form O-glucuronide conjugates. The highlighted functional group indicates the location of conjugation. Dashed circles demonstrate the possible location of conjugation however, the exact, known, location is not reported.

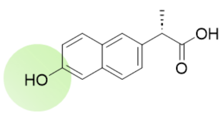

O-desmethyl naproxen

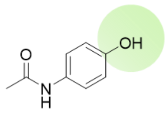

acetaminophen

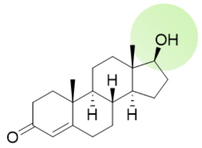

testosterone

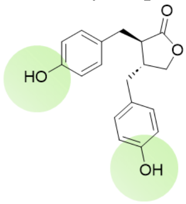

Enterolactone

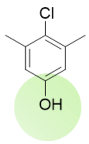

chloroxylenol

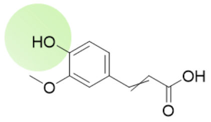

Ferulic acid

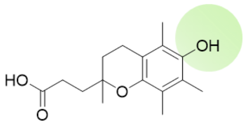

a-CEHC

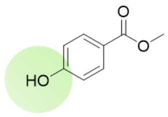

methylparaben

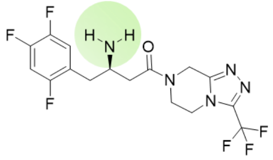

Sitagliptin (N-sulfate)

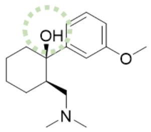

Tramadol (proposed)

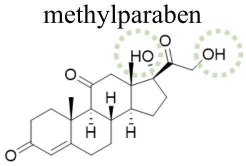

Cortisone (proposed)

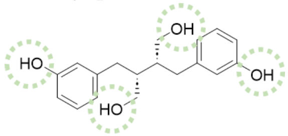

Enterodiol (proposed)

Figure S4. Chemical targets under study that form sulfate conjugates. The highlighted functional group indicates the location of conjugation. Dashed circles demonstrate the possible location of conjugation however, the exact, known, location is not reported.

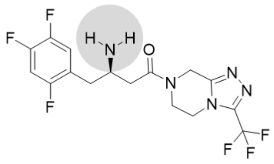

sitagliptin

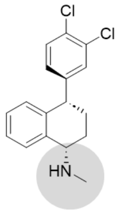

sertraline

Figure S5. Chemical targets under study that form N-carbamoyl conjugates. The highlighted functional group indicates the location of conjugation.

Table S4. Understanding the changes and increase in concentration of the free analyte in the control and following enzymatic deconjugation with β-Glucuronidase or arylsulfatase enzymes.

Class	Analyte	Control		β-Glucuronidase	arylsulfatase
		Maximum Decrease in Concentration from T0 (%)	Maximum Increase in Concentration from T0 (%)	Maximum Increase in Concentration from Control (%)	Maximum Increase in Concentration from Control (%)
Illicit drugs	Amphetamine*	-27.45	4.27	78.47	5.77
	Cocaine	-0.70	6.80	-0.59	1.41
	Benzoylecgonine*	-2.27	8.05	0.60	14.04
	Cocaethylene	-1.75	9.64	-2.70	-0.93
	Ketamine	-0.52	9.38	-1.34	4.71
	Norketamine	1.87	7.54	4.08	5.01
	Methamphetamine	-3.42	4.02	3.36	3.16
	MDMA	-14.06	0.00	11.26	6.55
Lifestyle	Caffeine*	3.67	14.89	0.91	10.55
	Paraxanthine*	-6.86	9.73	17.86	54.46
	Nicotine*	86.37	150.46	268.28	-1.93
	Cotinine‡	4.62	10.51	82.30	81.32
Pharmaceuticals	5-OH Lansoprazole	-5.12	25.93	-24.42	5.43
	Acetaminophen*	3.41	10.87	27.31	38.84
	Amitriptyline	-18.36	41.95	-33.54	-26.12
	Atenolol	-1.60	12.55	-7.59	-7.15
	Atorvastatin*	-28.08	0.00	57.50	13.61
	2-OH Atorvastatin*	-23.55	0.00	44.35	7.23
	Bezafibrate	-16.49	4.26	-1.34	-1.51
	Capecitabine*	-1.98	7.66	-8.12	67.09
	Carbamazepine‡	-6.86	8.49	10.88	15.14
	Citalopram‡	-4.33	0.42	10.09	10.50
	Desmethyl citalopram‡	32.80	178.43	106.44	151.29
	Codeine*	1.03	7.23	16.82	6.78
	Dihydrocodeine	4.56	14.09	3.87	2.87
	Diltiazem*	8.81	37.13	-26.95	58.77
	N-desmethyl diltiazem‡	102.49	221.45	132.30	54.57
	Fexofenadine	-52.42	42.05	-23.34	-11.55
	Fluoxetine‡	12.66	44.52	25.74	84.58
	Gabapentin*	-8.47	3.74	31.81	6.81
	Gliclazide*	-4.31	23.63	65.66	10.91
	OH-gliclazide	-11.84	23.45	-14.91	-5.55
	Ibuprofen	4.68	6.86	-5.72	-7.67
	2-OH ibuprofen	-14.92	22.21	1.49	14.17
	Levetiracetam	-28.47	0.00	-34.60	15.91
	Methadone	9.11	56.34	-9.11	-9.11
	EDDP	-5.40	5.52	3.38	3.80
	Memantine*	13.70	28.36	-9.37	25.64
	Mirtazapine‡	-1.24	7.83	48.54	49.96

Class	Analyte	Control		β-Glucuronidase	arylsulfatase
		Maximum Decrease in Concentration from T0 (%)	Maximum Increase in Concentration from T0 (%)	Maximum Increase in Concentration from Control (%)	Maximum Increase in Concentration from Control (%)
	Morphine‡	-8.63	4.75	67.00	34.02
	Naproxen	-7.19	6.57	2.46	-3.21
	O-desmethyl Naproxen‡	-10.03	21.60	59.75	46.91
	Nortriptyline	-56.50	0.00	0.00	-28.71
	10-OH nortriptyline*	-10.71	5.02	42.61	-11.87
	Pregabalin*	7.18	34.41	-8.63	23.45
	Propranolol‡	-9.47	29.12	67.02	75.64
	Quetiapine‡	3.64	30.54	37.98	28.02
	Sertraline‡	-18.80	31.62	19.15	13.52
	Sitagliptin	-8.51	7.45	-5.11	-13.08
	Tramadol*	-6.21	17.61	39.65	11.21
	O-desmethyl tramadol‡	-7.71	0.00	45.39	33.98
	N-desmethyl tramadol‡	-22.25	0.83	74.25	20.60
	Valsartan	-23.99	0.00	-12.87	-9.67
	Venlafaxine*	2.14	37.07	-27.90	32.98
	Desvenlafaxine*	-4.26	15.60	-9.76	43.12
Food	1-methyl-2-pyridone-5-carboxamide*	-20.57	0.00	42.91	0.00
	5-(3′,4′-Dihydroxyphenyl)-γ-valerolactone‡	-58.25	0.00	103.82	216.58
	4-Pyridoxic acid*	-13.51	42.14	67.73	-83.43
	Acesulfame K‡	-7.26	10.18	38.73	24.03
	α-CEHC	-6.96	22.85	-10.81	-15.16
	Enterodiol*	0.19	21.16	-4.31	23.49
	Enterolactone‡	11.13	38.13	51.86	62.99
	Ferulic acid‡	-8.55	91.84	297.49	222.04
	Riboflavin‡	-2.94	142.10	120.37	1551.59
	Saccharin	-4.20	27.86	5.53	9.03
	Sucralose	0.92	12.08	-14.30	9.75
	D,L-Sulforaphane-N-acetyl-L-cysteine	13.53	36.21	11.37	-3.65
Endogenous	2-deoxyguanosine‡	-15.76	314.58	220.24	1919.10
	Androstenedione‡	-46.47	-21.00	28.59	33.67
	Cortisone	2.14	9.18	1.63	8.44
	Hippuric acid	214.75	456.92	-113.82	-90.95
	HNE-MA	-19.25	-6.29	12.27	4.11
	Indoxyl Sulfate	0.56	15.54	-31.64	-102.69
	Phenyl acetyl glutamine	40.30	177.83	-164.58	-187.62
	Progesterone	-6.73	-3.40	0.39	-0.02
	Testosterone*	-14.78	28.23	-28.23	236.48
Personal care products	Benzophenone-4	0.80	12.65	-3.48	2.57
	Chloroxylenol‡	-18.24	-4.40	50.39	31.63
	Methylparaben‡	2.65	8.80	45.94	49.21

Class	Analyte	Control		β-Glucuronidase	arylsulfatase
		Maximum Decrease in Concentration from T0 (%)	Maximum Increase in Concentration from T0 (%)	Maximum Increase in Concentration from Control (%)	Maximum Increase in Concentration from Control (%)

* increase in concentration as per section 3.1 in one of two β-Glucuronidase or arylsulfatase enzymes.
‡ increase in concentration as per section 3.1 in both β-Glucuronidase or arylsulfatase enzymes.

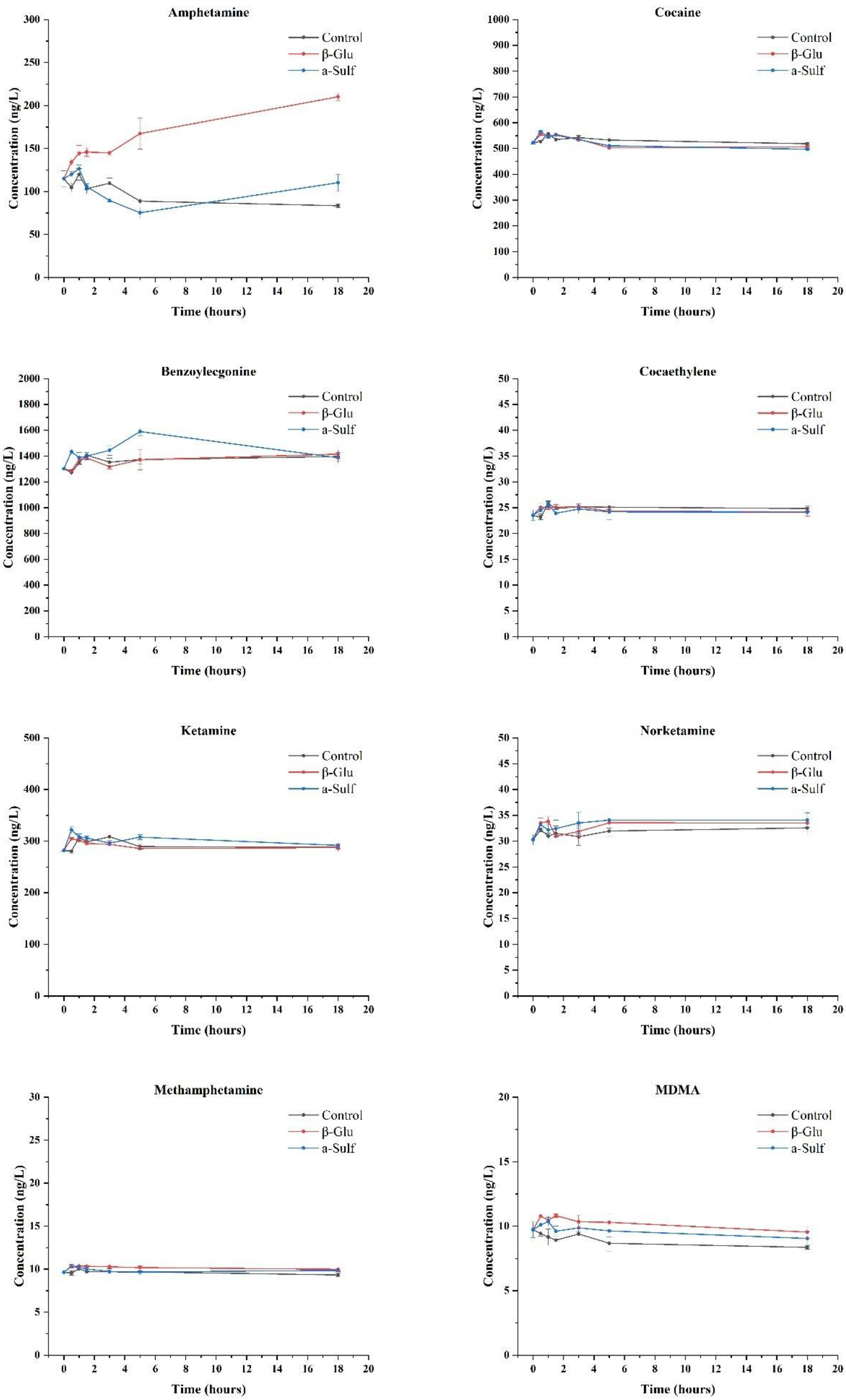

Figure S6. Time-concentration plots of 8 illicit drugs (amphetamine, cocaine, benzoylecgonine, cocaethylene, ketamine, norketamine, methamphetamine and MDMA) showing how the concentration of free analyte varies following enzymatic deconjugation with β-glucuronidase and arylsulfatase.

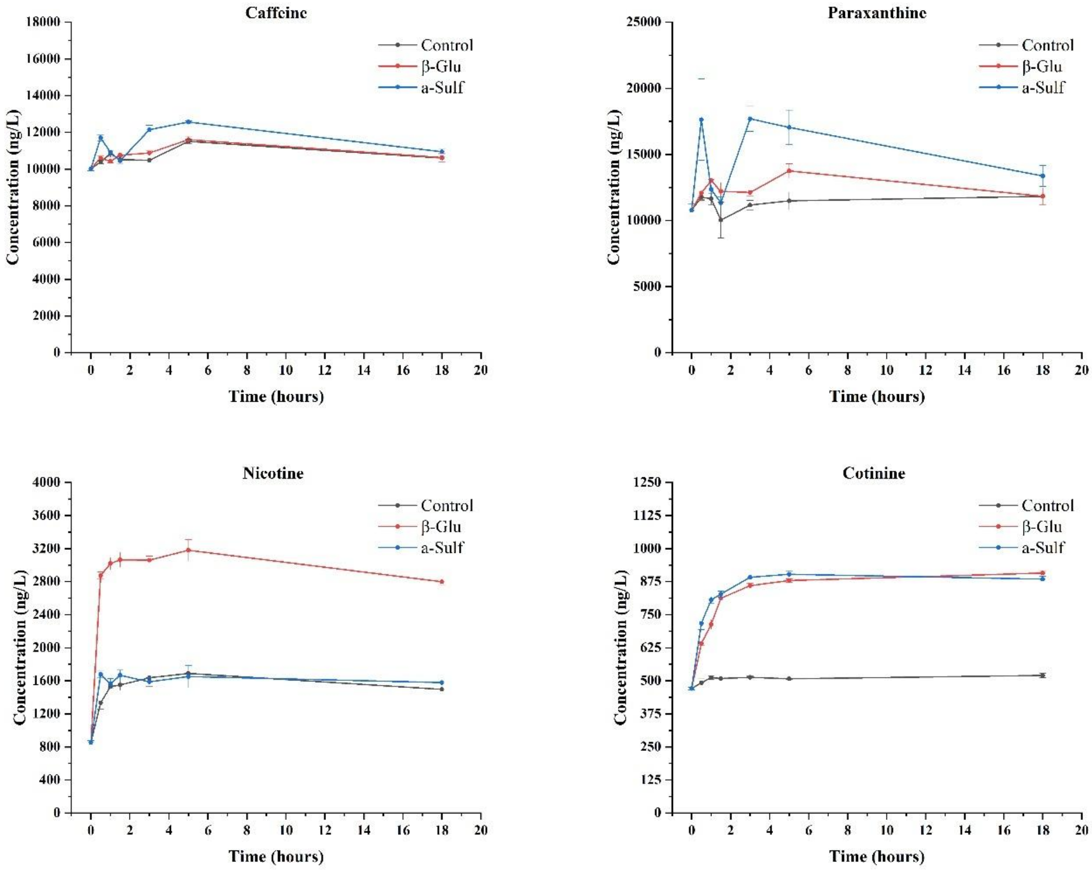

Figure S7. Time-concentration plots of 4 lifestyle chemicals (caffeine, paraxanthine, nicotine and cotinine) showing how the concentration of free analyte varies following enzymatic deconjugation with β-glucuronidase and arylsulfatase.

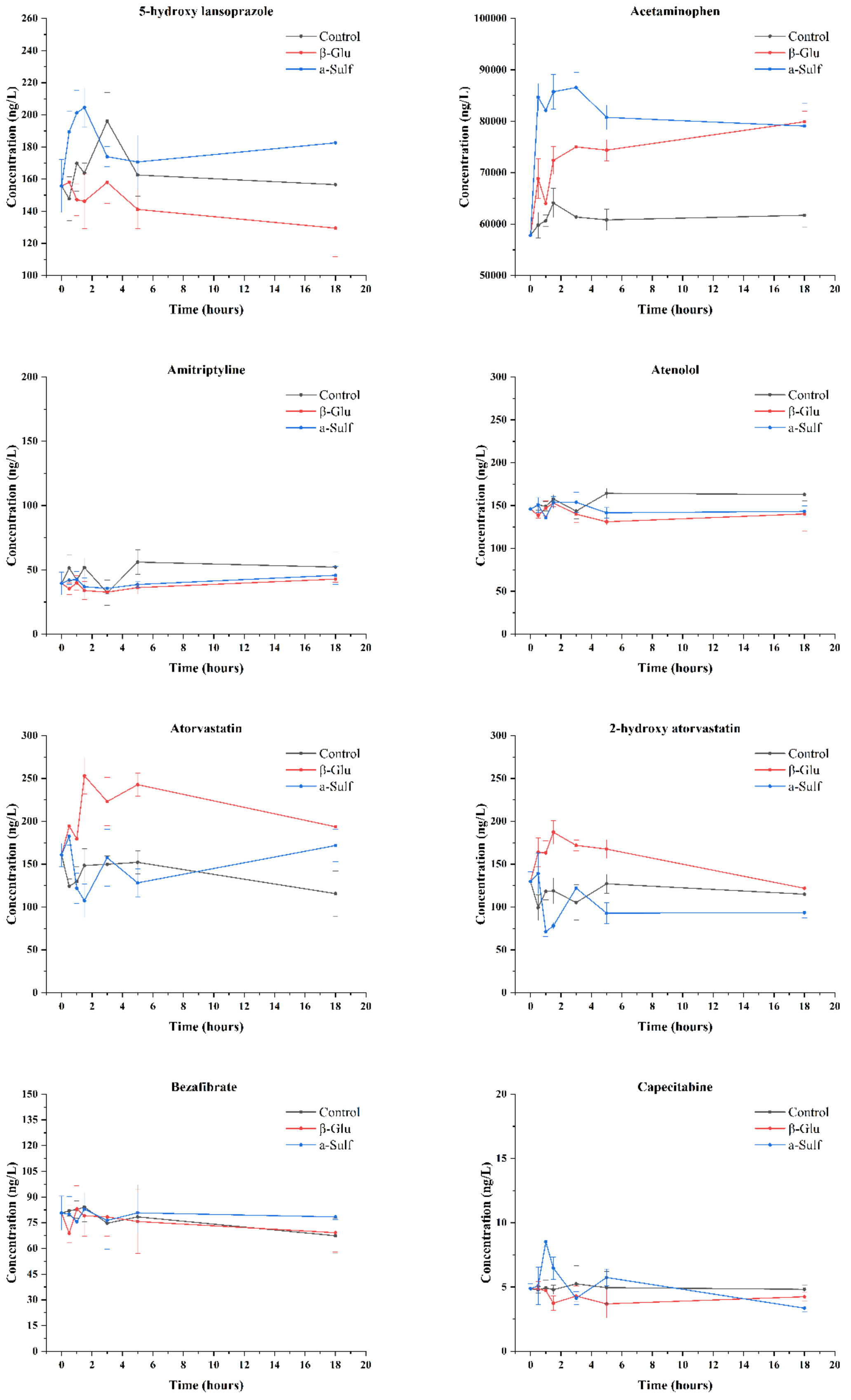

Figure S8. Time-concentration plots of 8 pharmaceuticals (5-OH lansoprazole, acetaminophen, amitriptyline, atenolol, atorvastatin, 2-OH atorvastatin, bezafibrate and capecitabine) showing how the concentration of free analyte varies following enzymatic deconjugation with β-glucuronidase and arylsulfatase.

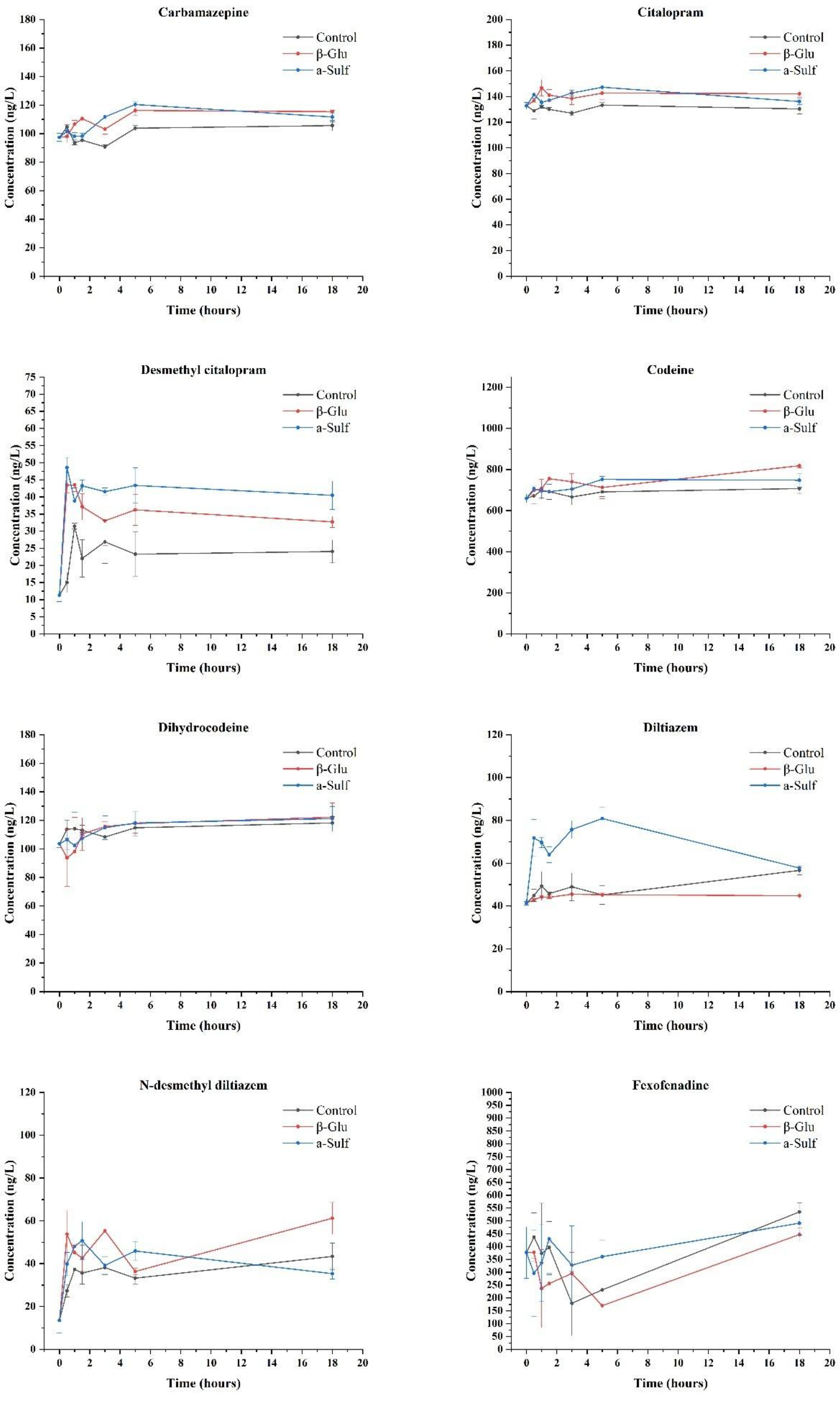

Figure S9. Time-concentration plots of 8 pharmaceuticals (carbamazepine, citalopram, desmethyl citalopram, codeine, dihydrocodeine, diltiazem, N-desmethyl diltiazem and fexofenadine) showing how the concentration of free analyte varies following enzymatic deconjugation with β-glucuronidase and arylsulfatase.

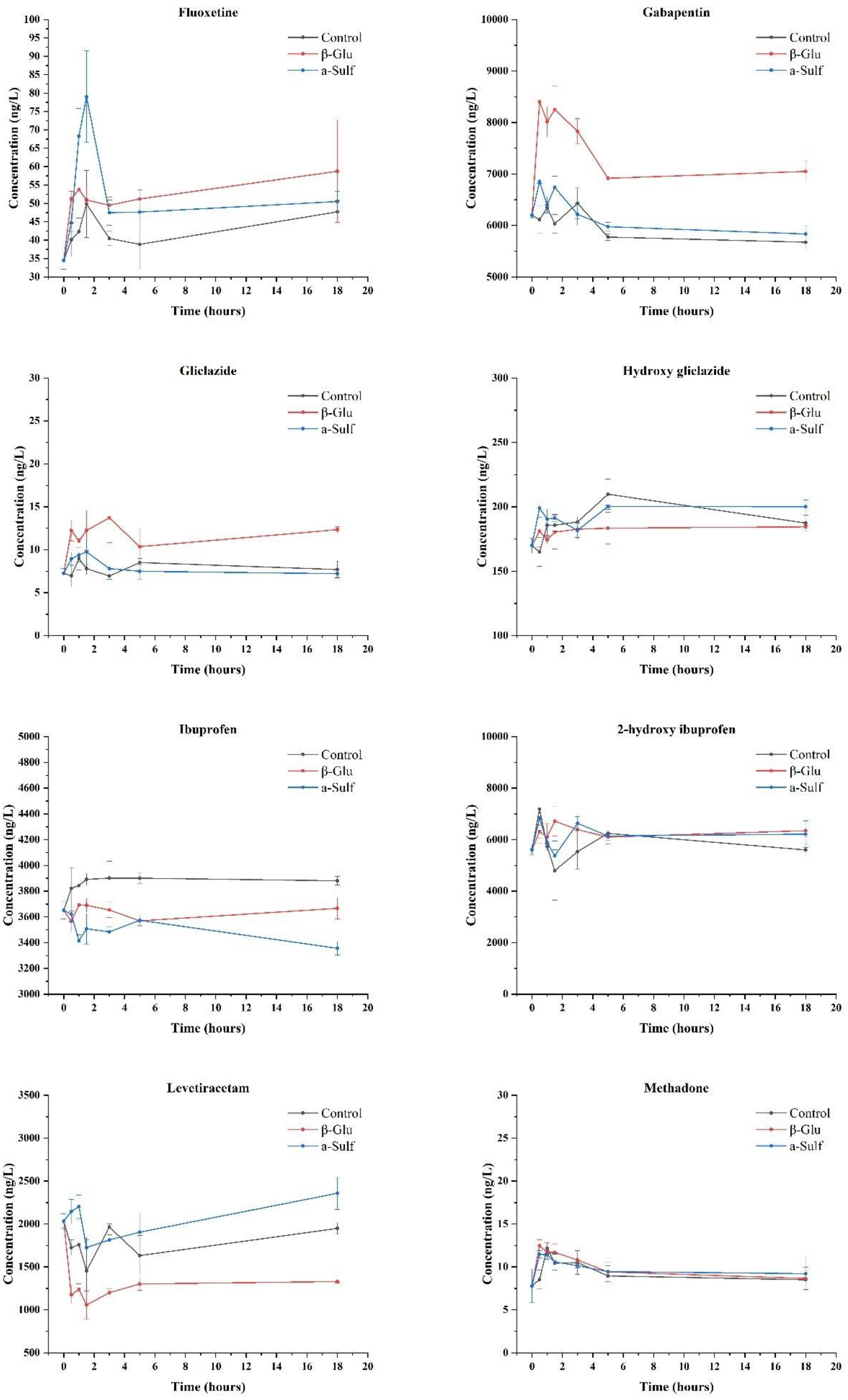

Figure S10. Time-concentration plots of 8 pharmaceuticals (fluoxetine, gabapentin, gliclazide, OH-gliclazide, ibuprofen, levetiracetam and methadone) showing how the concentration of free analyte varies following enzymatic deconjugation with β-glucuronidase and arylsulfatase.

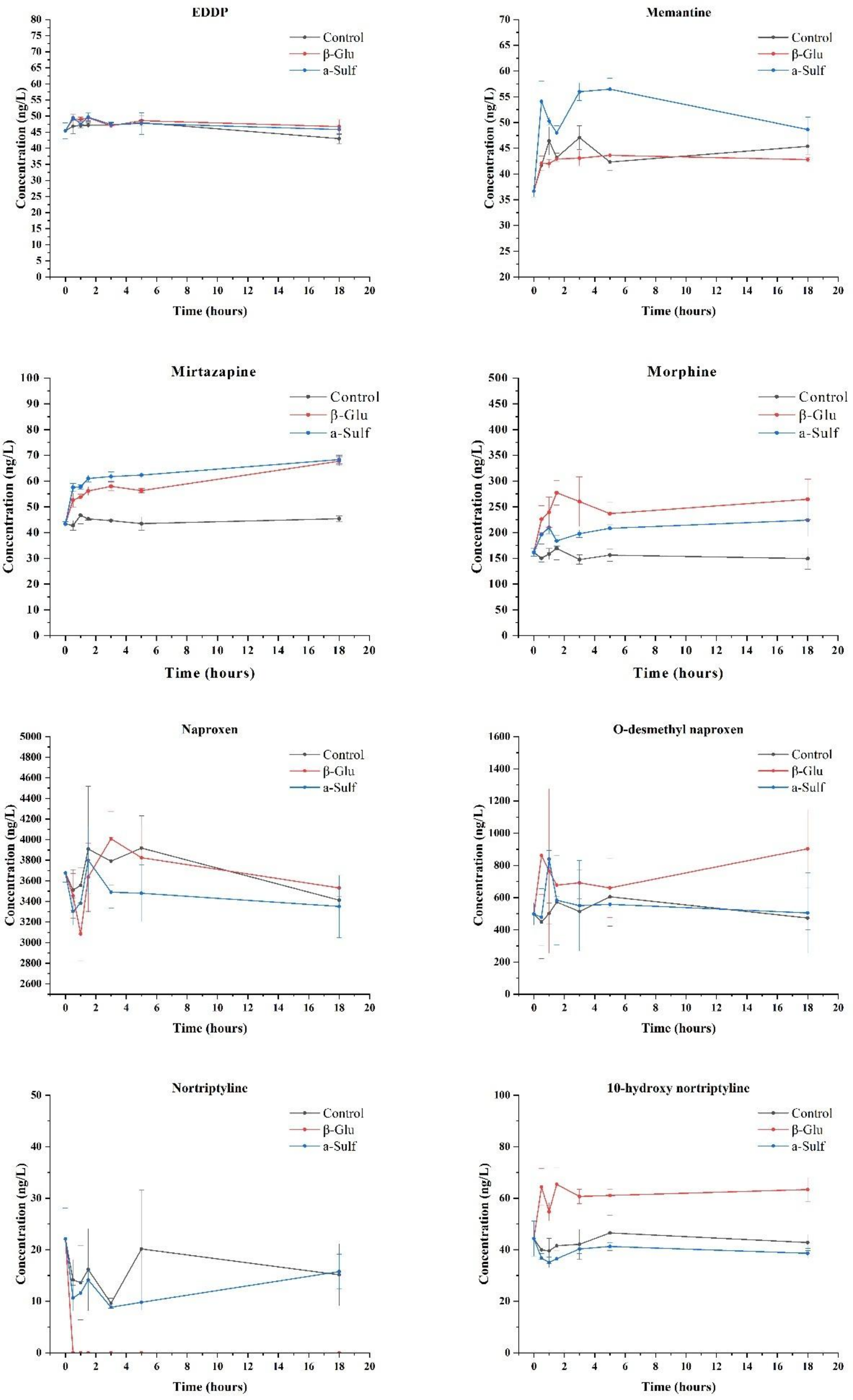

Figure S11. Time-concentration plots of 8 pharmaceuticals (EDDP, memantine, mirtazapine, morphine, naproxen, O-desmethyl naproxen, nortriptyline, 10-OH nortriptyline) showing how the concentration of free analyte varies following enzymatic deconjugation with β-glucuronidase and arylsulfatase.

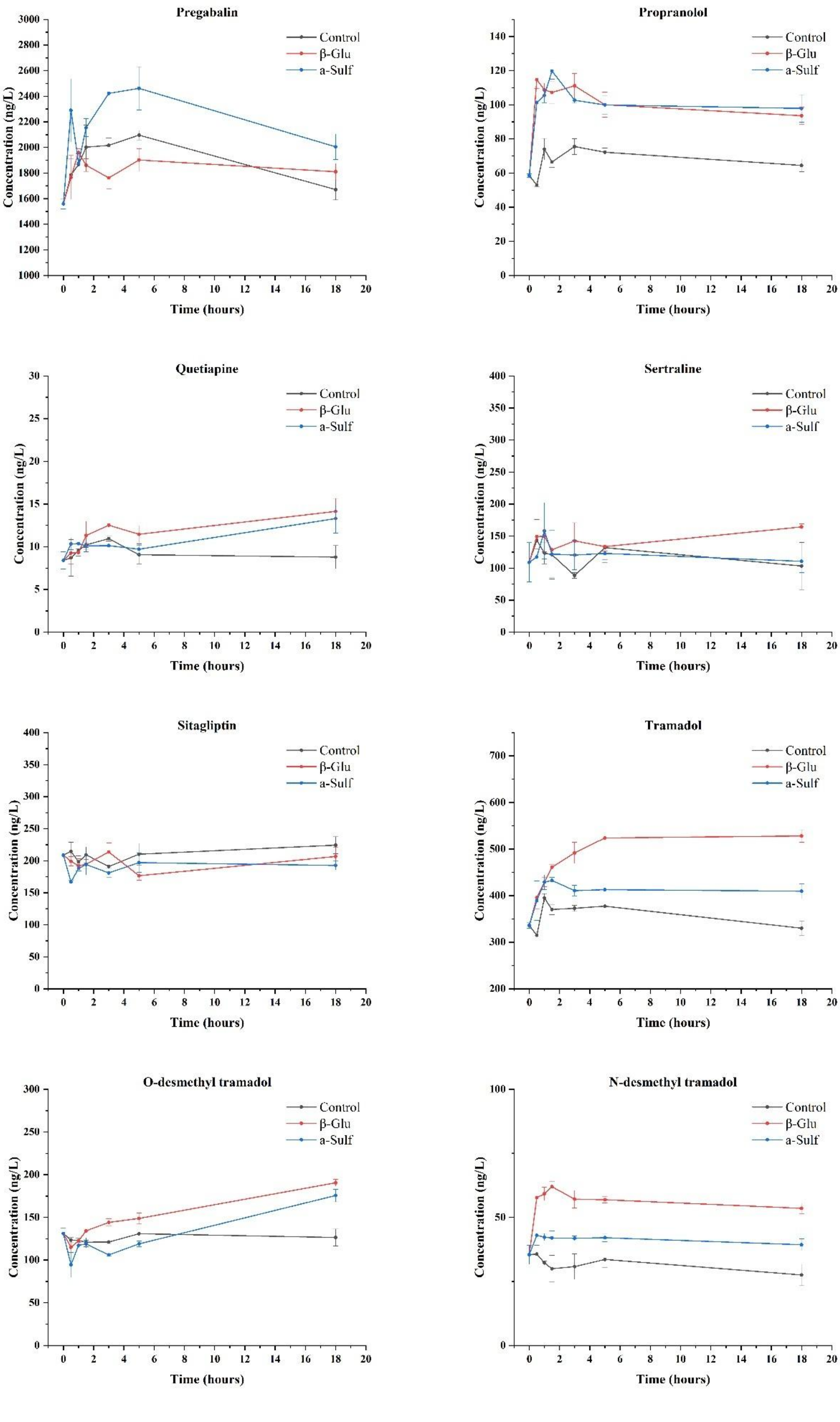

Figure S12. Time-concentration plots of 8 pharmaceuticals (pregabalin, propranolol, quetiapine, sertraline, sitagliptin, tramadol, O-desmethyl tramadol, N-desmethyl tramadol) showing how the concentration of free analyte varies following enzymatic deconjugation with β-glucuronidase and arylsulfatase.

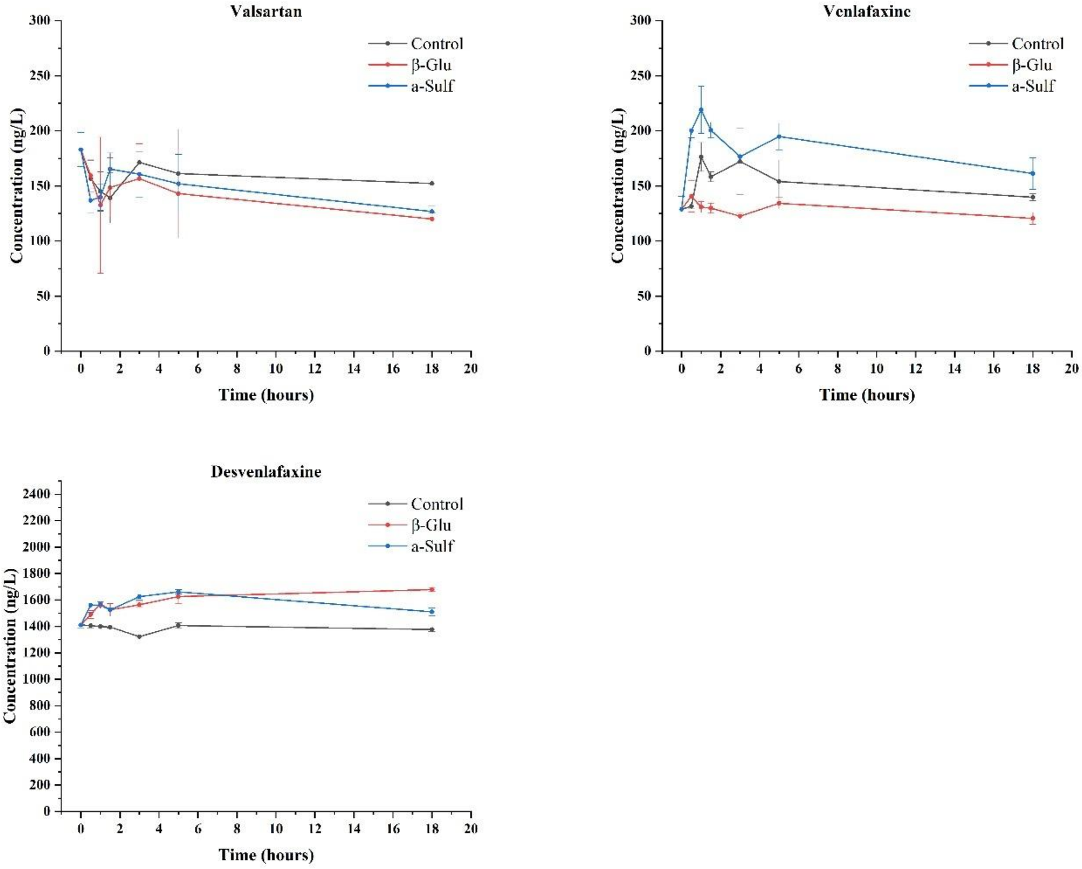

Figure S13. Time-concentration plots of 3 pharmaceuticals (valsartan, venlafaxine, desvenlafaxine) showing how the concentration of free analyte varies following enzymatic deconjugation with β-glucuronidase and arylsulfatase.

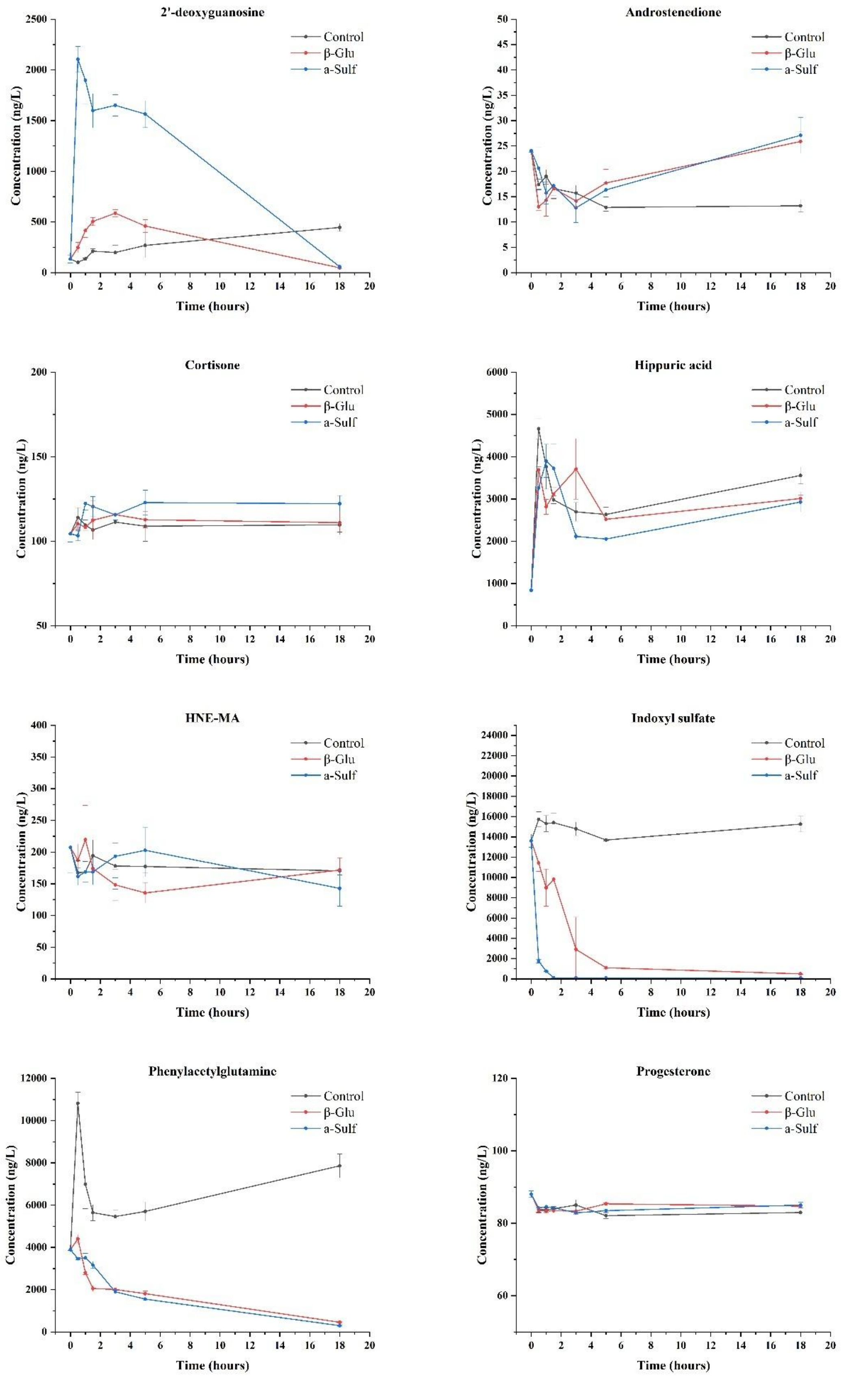

Figure S14. Time-concentration plots of 8 human markers (2’-deoxyguanosine, androstenedione, cortisone, hippuric acid, HNE-MA, indoxyl sulfate, phenylacetylglutamine and progesterone) showing how the concentration of free analyte varies following enzymatic deconjugation with β-glucuronidase and arylsulfatase.

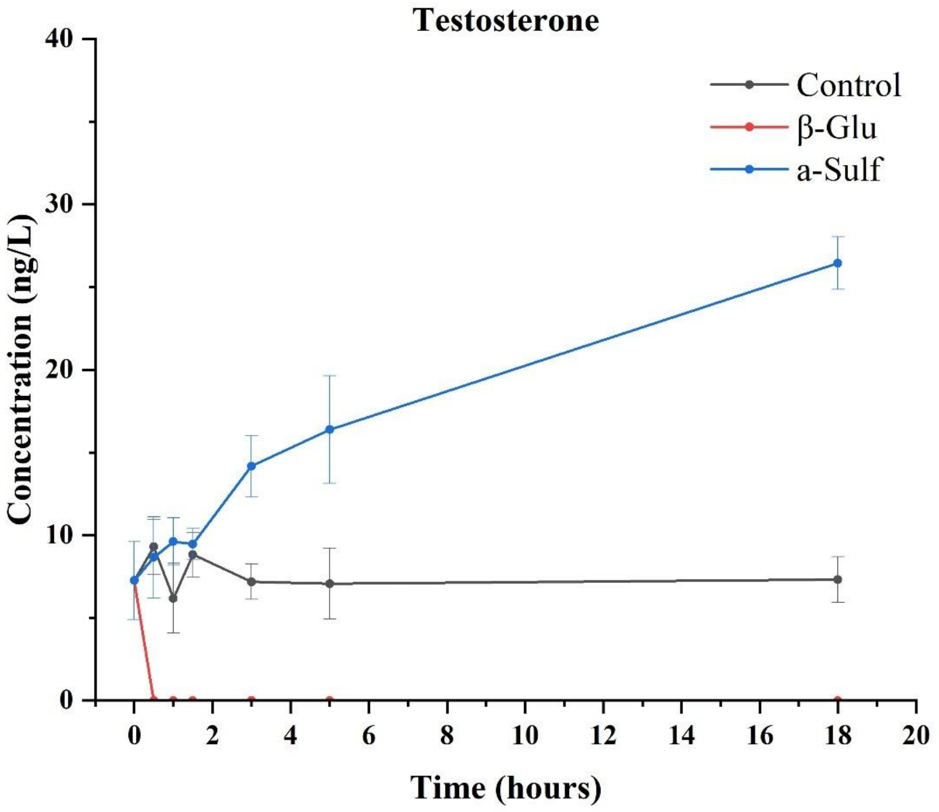

Figure S15. Time-concentration plots of testosterone showing how the concentration of free analyte varies following enzymatic deconjugation with β-glucuronidase and arylsulfatase.

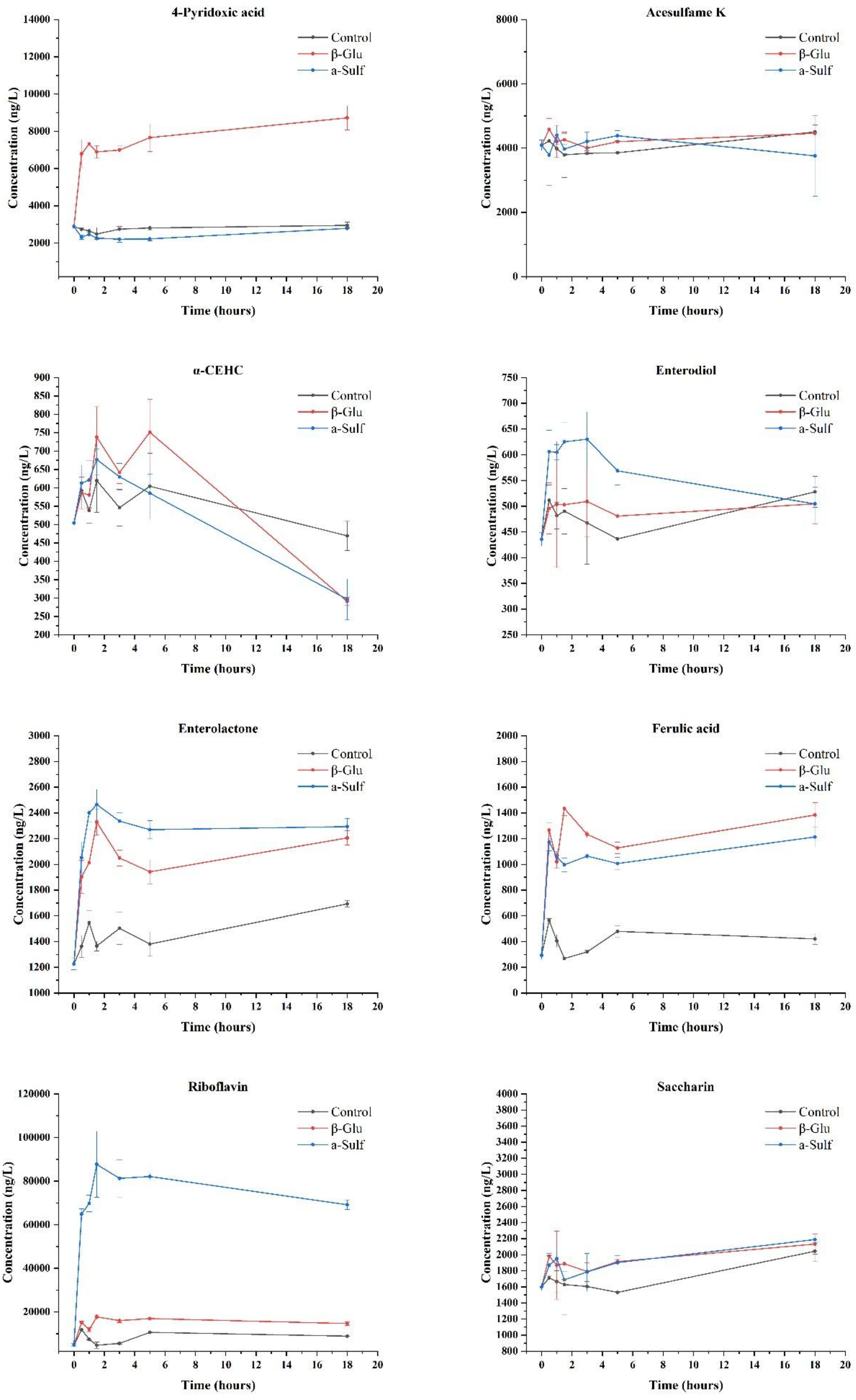

Figure S16. Time-concentration plots of 8 food markers (4-pyridoxic acid, acesulfame K, a-CEHC, enterodiol, enterolactone, ferulic acid, riboflavin and saccharin) showing how the concentration of free analyte varies following enzymatic deconjugation with β-glucuronidase and arylsulfatase.

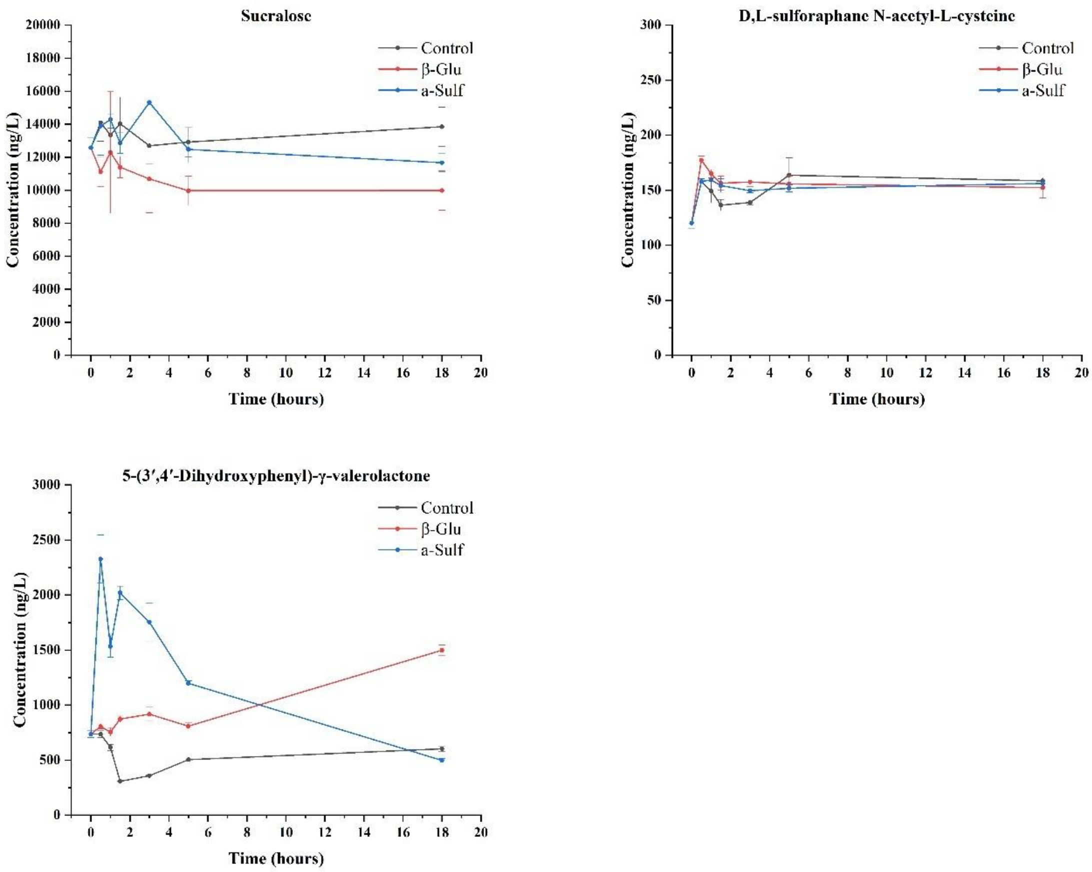

Figure S17. Time-concentration plots of 3 food markers (sucralose and D,L-sulforaphane N-acetyl-L-cysteine, 5-DHPV) showing how the concentration of free analyte varies following enzymatic deconjugation with β-glucuronidase and arylsulfatase.

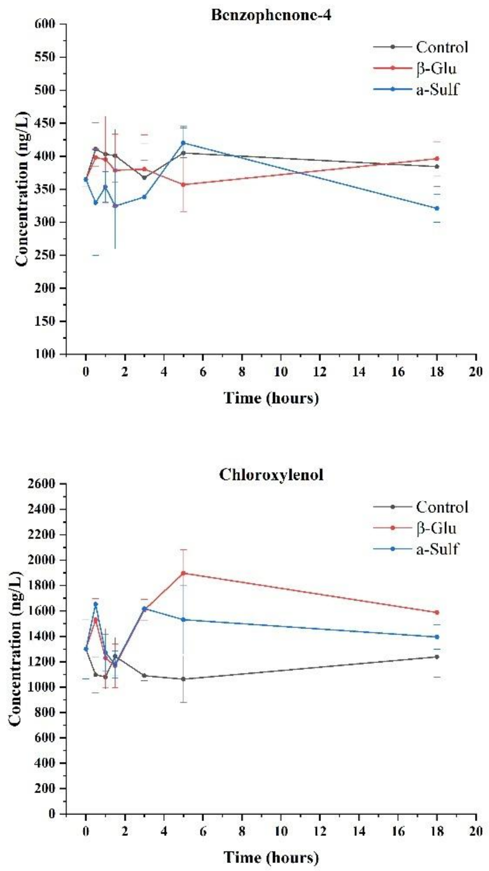

Figure S18. Time-concentration plots of 3 Personal care products (benzophenone-4, methylparaben and chloroxylenol) showing how the concentration of free analyte varies upon enzymatic deconjugation with β-glucuronidase and arylsulfatase.

Table S5. Indication of concentration increase following enzymatic deconjugation and if the concentration had plateaued, potentially indicating complete deconjugation. The data plateau was determined to be significant following the Mann-Kendall test.

Analyte	Class	Increased concentration of free analyte? (relative to control+ T0)		Conjugation observed in literature?	Concentration plateau?		Comments
		β-Glucuronidase	arylsulfatase		β-Glucuronidase	arylsulfatase	
Amphetamine	Illicit drug	✓	✗	✗	✗ (p = 0.0069)	✓ (p = 0.23)	
Cocaine		✗	✗	✗	✓ (p = 0.23)	✓ (p = 0.13)	
Benzoylecgonine		✗	✗	✗	✓ (p = 0.13)	✓ (p = 0.37)	
Cocaethylene		✗	✗	✗	✓ (p = 0.76)	✓ (p = 1.00)	
Ketamine		✗	✗	✗	✓ (p = 0.37)	✓ (p = 0.55)	
Norketamine		✗	✗	✗	✓ (p = 0.37)	✗ (p = 0.016)	The increasing concentration of norketamine (arylsulfatase) is insignificant
Methamphetamine		✗	✗	✗	✓ (p = 0.55)	✓ (p = 0.55)	
MDMA		✗	✗	✗	✓ (p = 0.37)	✓ (p = 0.23)	
Caffeine	Lifestyle	✗	✓	✗	✓ (p = 0.072)	✓ (p = 0.23)	
Paraxanthine		✓	✓	✗	✓ (p = 0.55)	✓ (p = 0.55)	
Nicotine		✓	✗	✓	✓ (p = 0.55)	✓ (p = 1.00)	
Cotinine		✓	✓	✓	✗ (p = 0.0027)	✗ (p = 0.016)	
5-OH Lansoprazole	Pharmaceutical	✗	✗	✓	✓ (p = 0.072)	✓ (p = 1.00)	
Acetaminophen		✓	✓	✓	✗ (p = 0.016)	✓ (p = 1.00)	
Amitriptyline		✗	✗	✓	✓ (p = 1.00)	✓ (p = 1.00)	
Atenolol		✗	✗	✓	✓ (p = 0.76)	✓ (p = 1.00)	
Atorvastatin		✓	✓	✓	✓ (p = 0.37)	✓ (p = 1.00)	
2-OH Atorvastatin		✓	✗	✓	✓ (p = 1.00)	✓ (p = 0.76)	

Bezafibrate	✗	✗	✓	✓ (p = 0.23)	✓ (p = 0.76)	
Capecitabine	✗	✓	✗	✗ (p = 0.035)	✓ (p = 0.55)	
Carbamazepine	✓	✓	✓	✗ (p = 0.035)	✓ (p = 0.072)	
Citalopram	✓	✓	✓	✓ (p = 0.23)	✓ (p = 0.23)	
Desmethyl citalopram	✓	✓	possible	✓ (p = 0.55)	✓ (p = 0.76)	
Codeine	✓	✓	✓	✗ (p = 0.035)	✓ (p = 0.13)	
Dihydrocodeine	✗	✗	✓	✗ (p = 0.016)	✗ (p = 0.016)	
Diltiazem	✗	✓	✗	✓ (p = 0.072)	✓ (p = 0.55)	
N-desmethyl diltiazem	✓	✓	✗	✓ (p = 0.37)	✓ (p = 1.00)	Sulfatase then decrease near end, initial increase of control
Fexofenadine	✗	✗	✗	✓ (p = 1.00)	✓ (p = 0.37)	
Fluoxetine	✓	✓	✓	✓ (p = 0.37)	✓ (p = 0.23)	
Gabapentin	✓	✗	✗	✓ (p = 0.55)	✓ (p = 0.13)	
Gliclazide	✓	✗	✗	✓ (p = 0.23)	✓ (p = 0.55)	
OH-Gliclazide	✗	✗	✗	✗ (p = 0.016)	✓ (p = 0.23)	
Ibuprofen	✗	✗	✓	✓ (p = 1.00)	✓ (p = 0.13)	
2-OH ibuprofen	✗	✗	✓	✓ (p = 0.55	✓ (p = 0.76)	
Levetiracetam	✗	✓	✗	✓ (p = 0.76)	✓ (p = 0.76)	
Methadone	✗	✗	✗	✓ (p = 0.23)	✓ (p = 0.23)	
EDDP	✗	✗	✗	✓ (p = 0.76)	✓ (p = 1.00)	
Memantine	✗	✓	✓	✓ (p = 0.072)	✓ (p = 0.37)	
Mirtazapine	✓	✓	✓	✗ (p = 0.0069)	✗ (p = 0.0027)	
Morphine	✓	✓	✓	✓ (p = 0.13)	✓ (p = 0.072)	
Naproxen	✗	✗	✓	✓	✓	

					(p = 0.76)	(p = 0.76)	
O-desmethyl naproxen		✓	✗	✓	✓ (p = 0.76)	✓ (p = 1.00)	
Nortriptyline		✗	✗	✗	✓ (p = 0.21)	✓ (p = 0.76)	
10-OH nortriptyline		✓	✗	✓	✓ (p = 0.37)	✓ (p = 1.00)	
Pregabalin		✗	✓	✗	✓ (p = 0.55)	✓ (p = 0.23)	
Propranolol		✓	✓	✓	✓ (p = 0.55)	✓ (p = 1.00)	
Quetiapine		✓	✓	✓	✗ (p = 0.0069)	✓ (p = 0.55)	
Sertraline		✓	✗	✓	✓ (p = 0.37)	✓ (p = 0.76)	
Sitagliptin		✗	✗	✓	✓ (p = 0.76)	✓ (p = 1.00)	
Tramadol		✓	✓	✓	✗ (p = 0.0027)	✓ (p = 0.55)	
O-desmethyl tramadol		✓	✓	✓	✗ (p = 0.0016)	✓ (p = 0.37)	
N-desmethyl tramadol		✓	✓	✗	✓ (p = 0.76)	✓ (p = 0.55)	
Valsartan		✗	✗	✓	✓ (p = 0.072)	✓ (p = 0.37)	
Venlafaxine		✗	✓	✗	✓ (p = 0.37)	✓ (p = 0.76)	
Desvenlafaxine		✓	✓	✓	✓ (p = 0.072)	✓ (p = 0.23)	
2'-deoxyguanosine	Human markers	-	-	-	✓ (p = 0.55)	✓ (p = 0.23)	Control has not plateaued (p = 0.016)
Androstenedione		✗	✗	✗	✓ (p = 0.37)	✓ (p = 0.76)	
Cortisone		✓	✗	✓	✓ (p = 0.13)	✓ (p = 0.23)	
Hippuric acid		✗	✗	-	✓ (p = 0.76)	✓ (p = 1.00)	
HNE-MA		✗	✗	-	✓ (p = 0.072)	✓ (p = 1.00)	
Indoxyl sulfate		-	-	-	✗ (p = 0.0069)	✗ (p = 0.016)	Sulfate therefore decreased
Phenyl acetyl glutamine		✗	✗	-	✗ (p = 0.0069)	✗ (p = 0.0069)	
Progesterone		✗	✗	✗	✓ (p = 0.76)	✓ (p = 0.55)	

Testosterone		✗	✓	✓	-	✗ (p = 0.0069)	β-glucuronidase below limit of quantification
4-Pyridoxic acid	Food	✗	✓	-	✓ (p = 0.072)	✓ (p = 0.76)	
Acesulfame K		✗	✗	✗	✓ (p = 1.00)	✓ (p = 1.00)	
a-CEHC		✓	✓	✓	✓ (p = 0.55)	✓ (p = 1.00)	Initial concentration increase, followed by degradation of analyte
Enterodiol		✗	✓	✓	✓ (p = 0.23)	✓ (p = 1.00)	
Enterolactone		✓	✓	✓	✓ (p = 0.13)	✓ (p = 0.55)	
Ferulic Acid		✓	✓	✓	✓ (p = 0.37)	✓ (p = 0.37)	
Riboflavin		✓	✓	-	✓ (p = 0.37)	✓ (p = 0.23)	
Saccharin		✗	✗	✗	✓ (p = 0.23)	✓ (p = 0.13)	
Sucralose		✗	✗	✓	✗ (p = 0.035)	✓ (p = 0.55)	
D,L-Sulforaphane N acetyl L-cysteine		✗	✗	✗	✓ (p = 0.37)	✓ (p = 1.00)	
Benzophenone-4		✗	✗	-	✓ (p = 1.00)	✓ (p = 0.55)	
Chloroxylenol	Personal care Product	✓	✓	✓	✓ (p = 0.37)	✓ (p = 1.00)	
Methylparaben		✓	✓	✓	✓ (p = 0.76)	✓ (p = 0.55)	

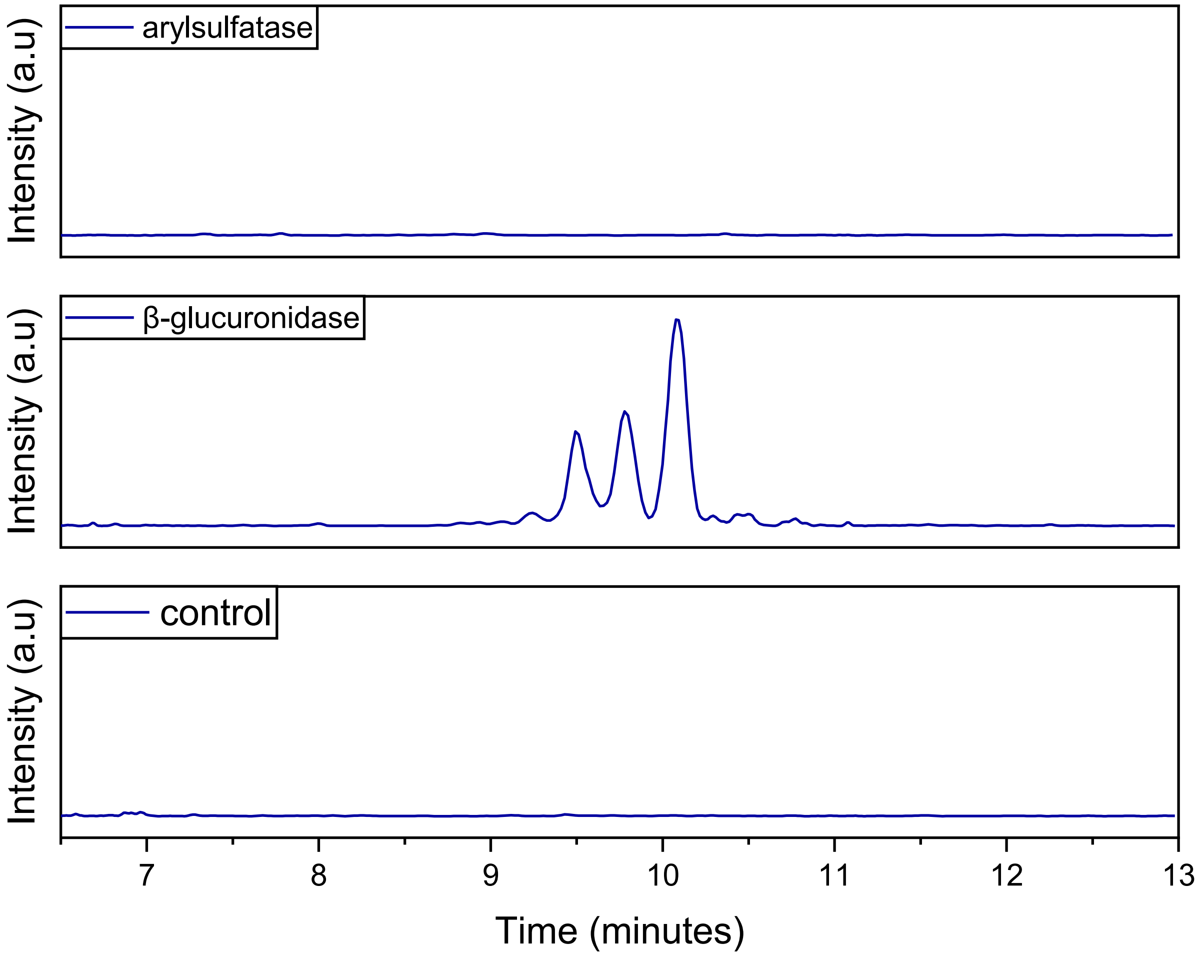

Figure S19. LC-MS/MS traces of 8-iso-PGF$_{2\beta}$ (353.4 → 193.2) in the control sample and following enzymatic deconjugation with β-glucuronidase and arylsulfatase.

Table S6. Concentration change (%) observed following 24 hours stored in bulk wastewater at room temperature.

Class	Analyte	Concentration change (%)	Reference
Illicit drugs	Amphetamine	2.1	[20]
	Cocaine	26.2	[20]
	Benzoylecgonine	7.5	[20]
	Cocaethylene	13.4	[20]
	Ketamine	5.8	[20]
	Norketamine	8.9	[20]
	Methamphetamine	51.4	[20]
	MDMA	24.9	[20]
Lifestyle	Caffeine	22.1	[20]
	Paraxanthine	12.8	[20]
	Nicotine	16.6	[20]
	Cotinine	10.3	[20]
Pharmaceuticals	5-OH Lansoprazole	94.8	[20]
	Acetaminophen	30.6	[20]
	Amitriptyline	nd	This study
	Atenolol	4.4	[20]
	Atorvastatin	36.1	[20]
	2-OH Atorvastatin	66.6	[20]
	Bezafibrate	1.5	[20]
	Capecitabine	nd	This study
	Carbamazepine	5.6	[20]
	Citalopram	6.7	[20]
	Desmethyl citalopram	5.1	[20]
	Codeine	2.1	[20]
	Dihydrocodeine†	2.3	[20]
	Diltiazem	3.9	[20]
	N-desmethyl diltiazem	36.9	[20]
	Fexofenadine	28.7	[20]
	Fluoxetine	not detected	This study
	Gabapentin	19.7	[20]
	Gliclazide	54.4	[20]
	OH-Gliclazide	40	[20]
	Ibuprofen	13.1	[20]
	2-OH ibuprofen	14.5	[20]
	Levetiracetam	44.5	This study
	Methadone	19.9	[20]
	EDDP	6.8	[20]
	Memantine	26.39	This study
	Mirtazapine	6.55	This study

	Morphine	6.87	This study
	Naproxen	5.1	[20]
	O-desmethyl naproxen	43.5	[20]
	Nortriptyline	nd	This study
	10-OH nortriptyline	nd	This study
	Pregabalin	9.2	[20]
	Propranolol	11.6	[20]
	Quetiapine	13.4	[20]
	Sertraline	31.11*	[10]
	Sitagliptin	10.5	[20]
	Tramadol	11.7	[20]
	O-desmethyl tramadol	14.8	[20]
	N-desmethyl tramadol	63.5	[20]
	Valsartan	16.81	This study
	Venlafaxine	43.8	[20]
	Desvenlafaxine	20.5	[20]
Endogenous	2'-deoxyguanosine	1.9	[20]
	Androstenedione	nd	This study
	Cortisone	42.1	This study
	Hippuric acid	94.1	This study
	HNE-MA	10	[79]
	Indoxyl sulfate	87.33	This study
	Phenyl acetyl glutamine	80.21	This study
	Progesterone	nd	This study
	Testosterone	nd	This study
Food	1-methyl-2-pyridone-5-carboxamide	14.1	This study
	4-Pyridoxic acid	17.5	This study
	Acesulfame K	17.3	This study
	a-CEHC	3.4	This study
	Enterodiol	34.1	This study
	Enterolactone	3.29	This study
	Ferulic Acid	nd	This study
	Riboflavin	28.22	This study
	Saccharin	24.04	This study
	Sucralose	21.55	This study
	D,L-Sulforaphane N acetyl L-cysteine	45.2	This study
Personal Care Products	Benzophenone-4	17.85	This study
	Chloroxylenol	22.97	This study
	Methylparaben	54.7	This study

nd = not detected
*12 hours rather than 24.

Table S7. Classification of sub-classes in Figure 7.

Class	Good Stability	Increase in concentration with β-Glucuronidase	Increase in concentration with arylsulfatase	Free Analyte forms a known conjugate	Description
I	✓	X	X	X	Only good stability observed
II	X	✓	X	X	Only Increase in concentration with β-Glucuronidase observed
III	X	X	✓	X	Only Increase in concentration with arylsulfatase observed
IV	X	X	X	✓	Only known conjugation occurs
V	✓	✓	X	X	Good stability & Increase in concentration with β-Glucuronidase observed
VI	X	✓	X	✓	Known conjugate & Increase in concentration with β-Glucuronidase observed
VII	X	X	✓	✓	Known conjugate & Increase in concentration with arylsulfatase observed
VIII	✓	X	✓	X	Good stability & Increase in concentration with arylsulfatase observed
IX	✓	X	X	✓	Good stability & known conjugate
X	X	✓	✓	✓	Known conjugate & Increase in conjugation with both enzymes
XI	✓	X	✓	✓	Good stability, known conjugate & Increase in concentration with arylsulfatase observed
XII	✓	✓	X	✓	Good stability, known conjugate & Increase in concentration with β-Glucuronidase observed
XIII	✓	✓	✓	X	Good stability & Increase in conjugation with both enzymes
XIV	X	✓	✓	X	Increase in conjugation with both enzymes
XV	✓	✓	✓	✓	Good stability, known conjugate & Increase in conjugation with both enzymes

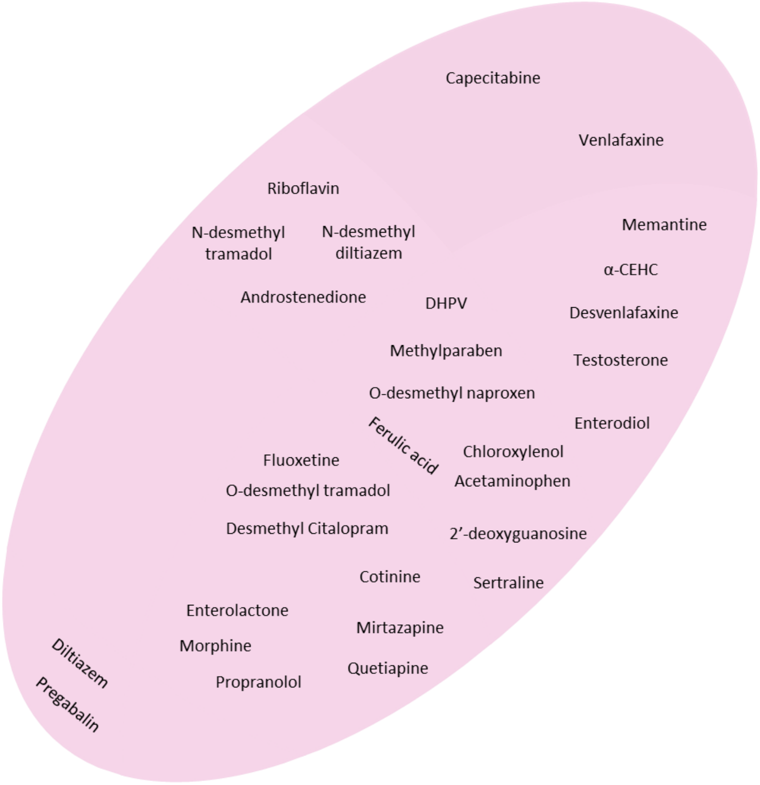

Figure S20. Supplementary to Figure 7b. All analytes which observed an increase in concentration following enzymatic deconjugation with arylsulfatase.

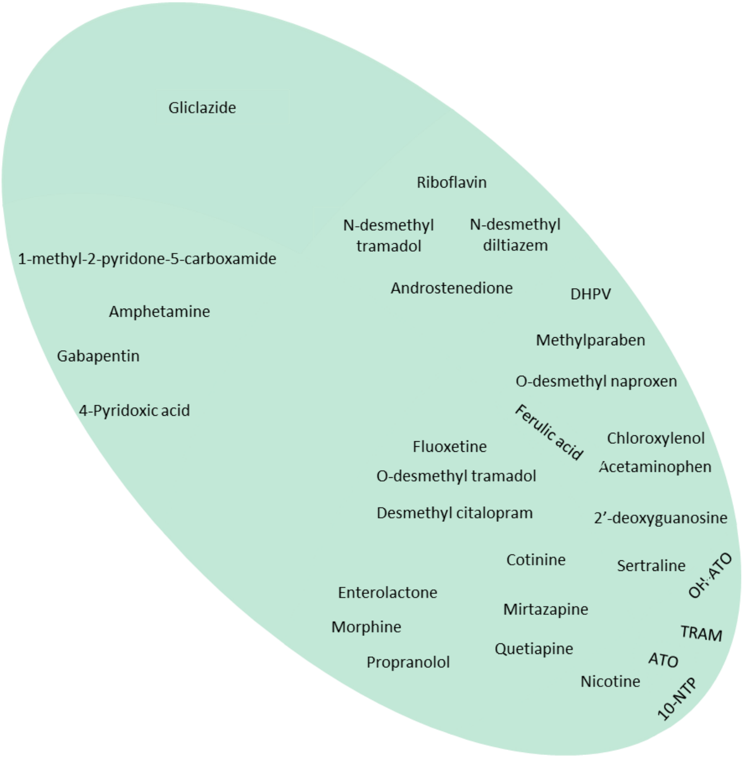

Figure S21. Supplementary to Figure 7b. All analytes which observed an increase in concentration following enzymatic deconjugation with β-glucuronidase.

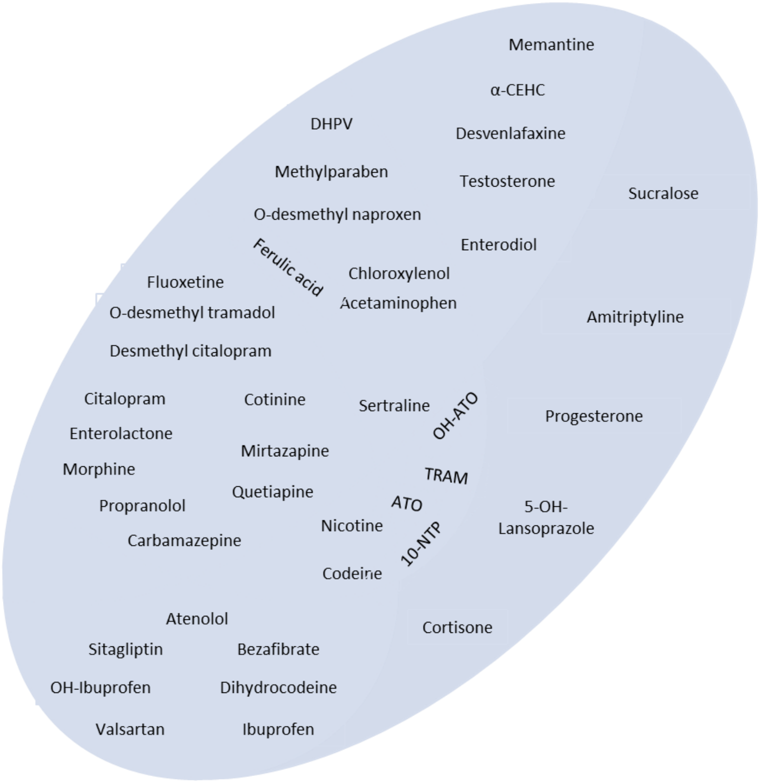

Figure S22. Supplementary to Figure 7b. All analytes which observed in the manuscript where the target analyte is known to form a conjugate.

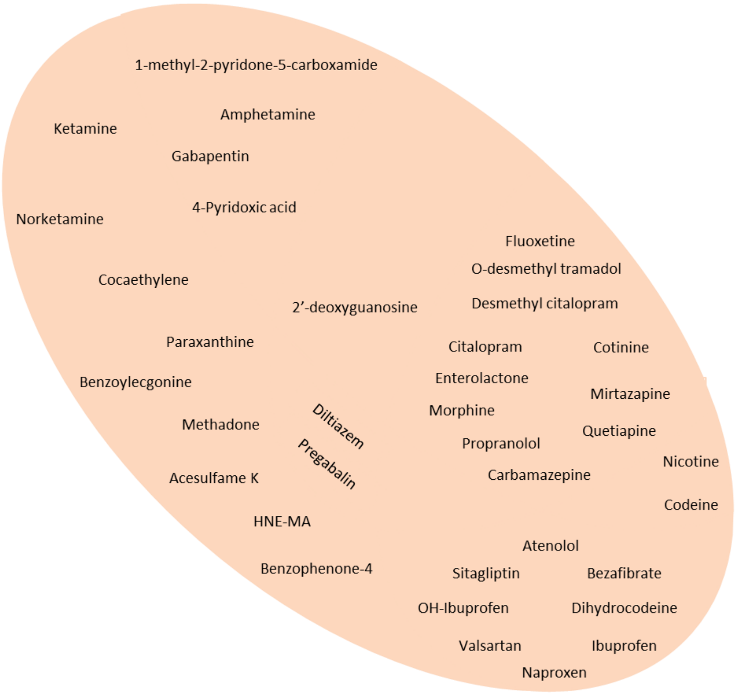

Figure S23. Supplementary to Figure 7b. All analytes which have good stability in wastewater.

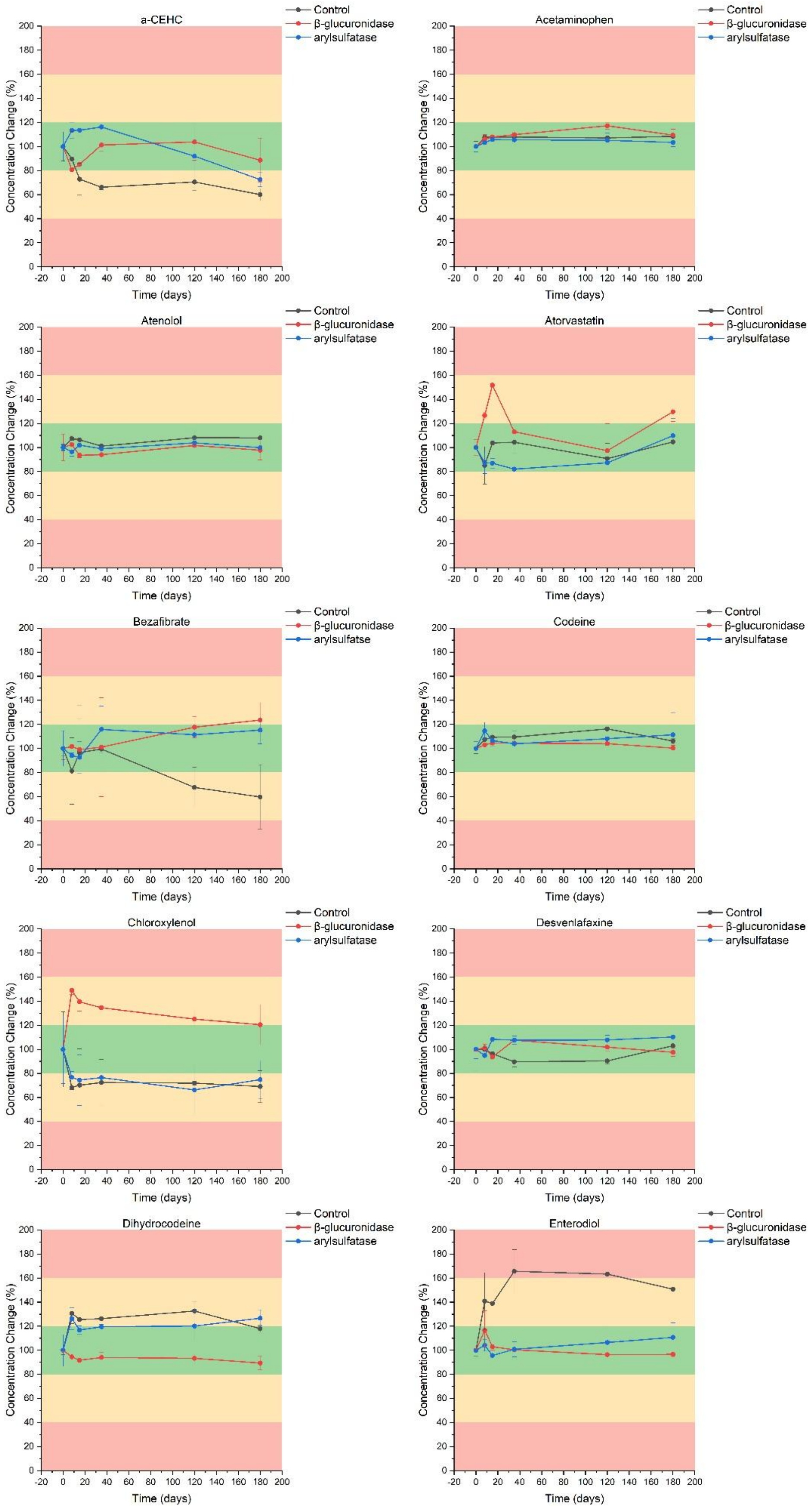

Figure S24. Time-concentration change plot of the in-freezer stability of 10 targets which are known to form phase II conjugates (Table S2).

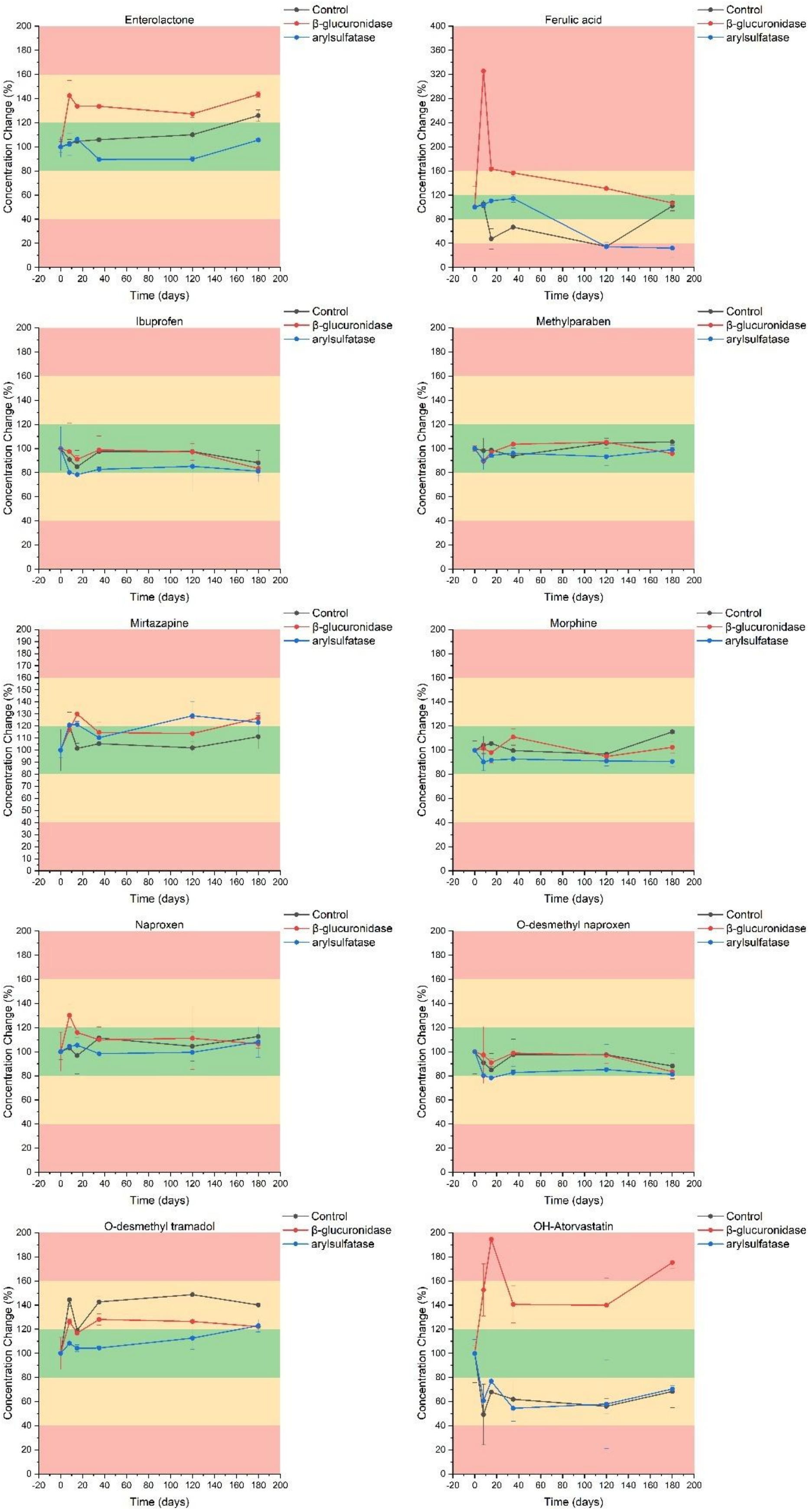

Figure S25. Time-concentration change plot of the in-freezer stability of 10 targets which are known to form phase II conjugates (Table S2).

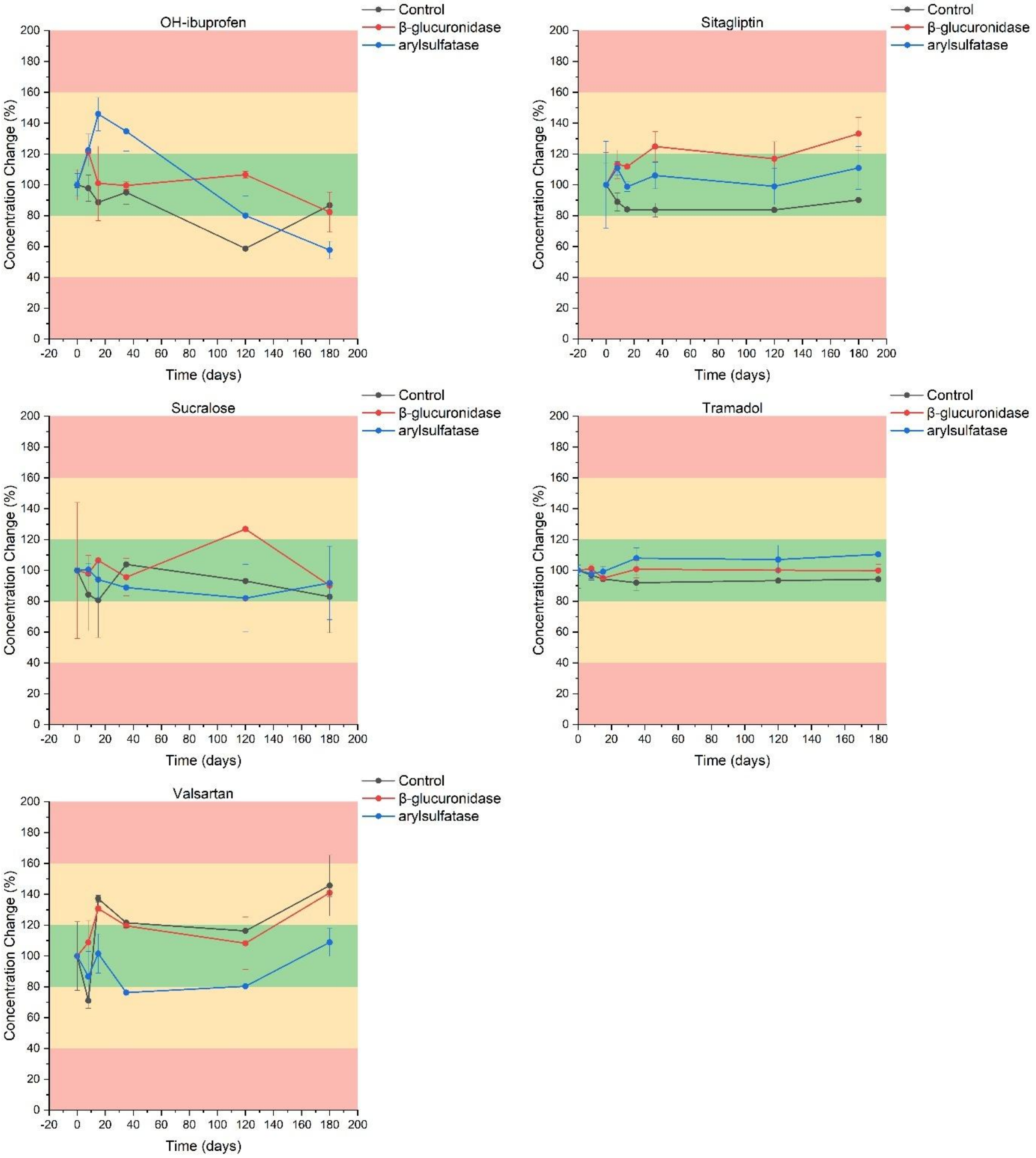

Figure S26. Time-concentration change plot of the in-freezer stability of 5 targets which are known to form phase II conjugates (Table S2).

References:

1. Markowitz, J.S. and K.S. Patrick, *The Clinical Pharmacokinetics of Amphetamines Utilized in the Treatment of Attention-Deficit/Hyperactivity Disorder.* Journal of child and adolescent psychopharmacology, 2017. **27**(8): p. 678-689.
2. Rice, J., et al., *Wastewater-based epidemiology combined with local prescription analysis as a tool for temporalmonitoring of drugs trends - A UK perspective.* The Science of the total environment, 2020. **735**: p. 139433-139433.
3. *Characterization of Differential Cocaine Metabolism in Mouse and Rat through Metabolomics-Guided Metabolite Profiling.* Drug metabolism and disposition/DMD online.
4. Roque Bravo, R., et al., *Cocaine: An Updated Overview on Chemistry, Detection, Biokinetics, and Pharmacotoxicological Aspects including Abuse Pattern.* Toxins, 2022. **14**(4): p. 278.
5. Gracia-Lor, E., E. Zuccato, and S. Castiglioni, *Refining correction factors for back-calculation of illicit drug use.* The Science of the total environment, 2016. **573**: p. 1648-1659.
6. González-Mariño, I., et al., *Spatio-temporal assessment of illicit drug use at large scale: evidence from 7 years of international wastewater monitoring.* Addiction (Abingdon, England), 2020. **115**(1): p. 109-120.
7. Laizure, S.C., et al., *Cocaethylene Metabolism and Interaction with Cocaine and Ethanol: Role of Carboxylesterases.* Drug metabolism and disposition : the biological fate of chemicals., 2003. **31**(1): p. 16-20.
8. Lavender, E., M. Hirasawa-Fujita, and E.F. Domino, *Ketamine's dose related multiple mechanisms of actions: Dissociative anesthetic to rapid antidepressant.* Behavioural brain research, 2020. **390**: p. 112631-112631.
9. Yargeau, V., et al., *Analysis of drugs of abuse in wastewater from two Canadian cities.* Science of the total environment, 2014. **487**: p. 722-730.
10. Baker, D.R., L. Barron, and B. Kasprzyk-Hordern, *Illicit and pharmaceutical drug consumption estimated via wastewater analysis. Part A: Chemical analysis and drug use estimates.* Science of the total environment, 2014. **487**(1): p. 629-641.
11. Caldwell, J., L.G. Dring, and R.T. Williams, *Metabolism of (14 C)methamphetamine in man, the guinea pig and the rat.* Biochemical journal, 1972. **129**(1): p. 11-22.
12. de la Torre, R., et al., *Human Pharmacology of MDMA.* Therapeutic Drug Monitoring, 2004. **26**(2): p. 137-144.
13. Caubet, M.-S., B. Comte, and J.-L. Brazier, *Determination of urinary 13C-caffeine metabolites by liquid chromatography–mass spectrometry: the use of metabolic ratios to assess CYP1A2 activity.* Journal of pharmaceutical and biomedical analysis, 2004. **34**(2): p. 379-389.
14. Gracia-Lor, E., et al., *Estimation of caffeine intake from analysis of caffeine metabolites in wastewater.* Science of the total environment, 2017. **609**: p. 1582-1588.
15. Benowitz, N.L., J. Hukkanen, and P. Jacob, *Nicotine Chemistry, Metabolism, Kinetics and Biomarkers.* Handbook of Experimental Pharmacology, 2009. **192**(192): p. 29-60.
16. Delhotal Landes, B., J.P. Petite, and B. Flouvat, *Clinical pharmacokinetics of lansoprazole.* Clinical pharmacokinetics, 1995. **28**(6): p. 458-470.
17. Kannan, A., et al., *The burden of city's pain treatment – A longitudinal one year study of two cities via wastewater-based epidemiology.* Water research (Oxford), 2023. **229**: p. 119391-119391.
18. Dahl-Puustinen, M.L., A. Åberg-Wistedt, and L. Bertilsson, *Glucuronidation of Amitriptyline in Man in Vivo.* Pharmacology & toxicology., 1989. **65**(1): p. 37-39.
19. Reeves, P.R., et al., *Metabolism of Atenolol in Man.* Xenobiotica, 1978. **8**(5): p. 313-320.

20. Ceolotto, N., et al., *A new Wastewater-Based Epidemiology workflow to estimate community wide non-communicable disease prevalence using pharmaceutical proxy data.* Journal of hazardous materials, 2024. **461**: p. 132645-132645.
21. Prueksaritanont, T., et al., *Glucuronidation of Statins in Animals and Humans: A Novel Mechanism of Statin Lactonization.* Drug metabolism and disposition : the biological fate of chemicals., 2002. **30**(5): p. 505-512.
22. Goosen, T.C., et al., *Atorvastatin Glucuronidation Is Minimally and Nonselectively Inhibited by the Fibrates Gemfibrozil, Fenofibrate, and Fenofibric Acid.* Drug metabolism and disposition : the biological fate of chemicals., 2007. **35**(8): p. 1315-1324.
23. Abshagen, U., et al., *Disposition pharmacokinetics of bezafibrate in man.* European journal of clinical pharmacology., 1979. **16**(1): p. 31-38.
24. Desmoulin, F., et al., *A Glucuronidation Pathway of Capecitabine Occurs in Rats but Not in Mice and Humans.* Drug Metabolism Letters, 2007. **1**(2): p. 101-107.
25. Judson, I.R., et al., *A human capecitabine excretion balance and pharmacokinetic study after administration of a single oral dose of 14C-labelled drug.* Investigational new drugs, 1999. **17**(1): p. 49-56.
26. Bahlmann, A., et al., *Carbamazepine and its metabolites in wastewater: Analytical pitfalls and occurrence in Germany and Portugal.* Water research (Oxford), 2014. **57**: p. 104-114.
27. Dalgaard, L. and C. Larsen, *Metabolism and excretion of citalopram in man: identification of O-acyl- and N-glucuronides.* Xenobiotica., 1999. **29**(10): p. 1033-1041.
28. Williams, D.G., D.J. Hatch, and R.F. Howard, *Codeine phosphate in paediatric medicine.* British journal of anaesthesia, 2001. **86**(3): p. 413-421.
29. Fromm, M.F., et al., *Dihydrocodeine: A new opioid substrate for the polymorphic CYP2D6 in humans.* Clinical pharmacology and therapeutics, 1995. **58**(4): p. 374-382.
30. Zisaki, A., L. Miskovic, and V. Hatzimanikatis, *Antihypertensive Drugs Metabolism: An Update to Pharmacokinetic Profiles and Computational Approaches.* Current pharmaceutical design, 2015. **21**(6): p. 806-822.
31. Yeung, P.K.F., et al., *Pharmacokinetics and metabolism of diltiazem in healthy males and females following a single oral dose.* European journal of drug metabolism and pharmacokinetics, 1993. **18**(2): p. 199-206.
32. Smith, S.M. and J.G. Gums, *Fexofenadine: biochemical, pharmacokinetic and pharmacodynamic properties and its unique role in allergic disorders.* Expert opinion on drug metabolism & toxicology, 2009. **5**(7): p. 813-822.
33. Altamura, A.C., A.R. Moro, and M. Percudani, *Clinical Pharmacokinetics of Fluoxetine.* Clinical pharmacokinetics., 1994. **26**(3): p. 201-214.
34. Lal, R.P., et al., *Clinical Pharmacokinetics of Gabapentin After Administration of Gabapentin Enacarbil Extended-Release Tablets in Patients With Varying Degrees of Renal Function Using Data From an Open-Label, Single-Dose Pharmacokinetic Study.* Clinical therapeutics, 2012. **34**(1): p. 201-213.
35. Oida, T., et al., *The metabolism of gliclazide in man.* Xenobiotica., 1985. **15**(1): p. 87-96.
36. Rudy, A.C., et al., *Stereoselective metabolism of ibuprofen in humans: administration of R-, S- and racemic ibuprofen.* The Journal of pharmacology and experimental therapeutics, 1991. **259**(3): p. 1133-1139.
37. De Logu, F., et al., *The acyl-glucuronide metabolite of ibuprofen has analgesic and anti-inflammatory effects via the TRPA1 channel.* Pharmacological research, 2019. **142**: p. 127-139.
38. Davies, N.M., *Clinical pharmacokinetics of ibuprofen : The first 30 years.* Clinical pharmacokinetics, 1998. **34**(2): p. 101-154.
39. Khetan, S.K. and T.J. Collins, *Human pharmaceuticals in the aquatic environment: A challenge to green chemisty.* Chemical reviews, 2007. **107**(6): p. 2319-2364.
40. Patsaios, P.N., *Clinical pharmacokinetics of levetiracetam.* Clinical pharmacokinetics, 2004. **43**(11): p. 707-724.

41. Ferrari, A., et al., *Methadone—metabolism, pharmacokinetics and interactions.* Pharmacological research, 2004. **50**(6): p. 551-559.
42. Thai, P.K., et al., *Refining the excretion factors of methadone and codeine for wastewater analysis — Combining data from pharmacokinetic and wastewater studies.* Environment international, 2016. **94**: p. 307-314.
43. Tampi, R.R. and C.H. van Dyck, *Memantine: efficacy and safety in mild-to-severe Alzheimer's disease.* Neuropsychiatric disease and treatment, 2007. **3**(2): p. 245-258.
44. Delbressine, L.P., et al., *Pharmacokinetics and biotransformation of mirtazapine in human volunteers.* Clinical drug investigation, 1998. **15**(1): p. 45-55.
45. HasslestrÖM, J. and J. SÄWe, *Morphine pharmacokinetics and metabolism in humans : enterohepatic cycling and relative contribution of metabolites to active opioid concentrations.* Clinical pharmacokinetics, 1993. **24**(4): p. 344-354.
46. Vree, T.B., M. van den Biggelaar-Martea, and C.P.W.G.M. Verwey-van Wissen, *Determination of naproxen and its metabolite O-desmethylnaproxen with their acyl glucuronides in human plasma and urine by means of direct gradient high-performance liquid chromatography.* Journal of chromatography. Biomedical applications, 1992. **578**(2): p. 239-249.
47. Jaggi, R., et al., *Conjugation of Desmethylnaproxen in the Rat—A Novel Acyl Glucuronide-Sulfate Diconjugate as a Major Biliary Metabolite.* Drug metabolism and disposition : the biological fate of chemicals., 2002. **30**(2): p. 161-166.
48. Breyer-Pfaff, U., *The Metabolic Fate of Amitriptyline, Nortriptyline and Amitriptylinoxide in Man.* Drug metabolism reviews, 2004. **36**(3-4): p. 723-746.
49. Baier-Weber, B., et al., *Glucuronides of hydroxylated metabolites of amitriptyline and nortriptyline isolated from rat bile.* Drug Metabolism and Disposition, 1988. **16**(3): p. 490-496.
50. Nordin, C. and L. Bertilsson, *Active Hydroxymetabolites of Antidepressants.* Clinical pharmacokinetics., 1995. **28**(1): p. 26-40.
51. Besag, F.M.C. and D. Berry, *Interactions between antiepileptic and antipsychotic drugs.* Drug safety, 2006. **29**(2): p. 95-118.
52. DeVane, C.L. and C.B. Nemeroff, *Clinical pharmacokinetics of quetiapine: an atypical antipsychotic.* Clinical pharmacokinetics, 2001. **40**(7): p. 509-522.
53. Tremaine, L.M., J.G. Stroh, and R.A. Ronfeld, *Characterization of a carbamic acid ester glucuronide of the secondary amine sertraline.* Drug Metabolism and Disposition, 1989. **17**(1): p. 58-63.
54. Murdoch, D. and D. McTavish, *Sertraline.* Drugs., 1992. **44**(4): p. 604-624.
55. Vincent, S.H., et al., *Metabolism And Excretion of the Dipeptidyl Peptidase 4 Inhibitor [14 C]Sitagliptin in Humans.* Drug metabolism and disposition : the biological fate of chemicals., 2007. **35**(4): p. 533-538.
56. Grond, S. and A. Sablotzki, *Clinical pharmacology of tramadol.* Clinical pharmacokinetics, 2004. **43**(13): p. 879-923.
57. Matsumoto, S., et al., *Selection of the candidate compound at an early stage of new drug development: retrospective pharmacokinetic and metabolic evaluations of valsartan using common marmosets.* Xenobiotica., 2022. **52**(6): p. 613-624.
58. Howell, S.R., et al., *Metabolic disposition of 14 C-venlafaxine in mouse, rat, dog, rhesus monkey and man.* Xenobiotica., 1993. **23**(4): p. 349-359.
59. Pasqualini, J.R., *Enzymes involved in the formation and transformation of steroid hormones in the fetal and placental compartments.* Journal of steroid biochemistry and molecular biology, 2005. **97**(5): p. 401-415.
60. Stančáková, A., et al., *The Excretion of Free Cortisol, Cortisone, Cortisol Sulfate and Cortisone Sulfate in Peripheral Vascular Disease, Diabetes Mellitus and Hyperthyroidism.* Hormone and Metabolic Research, 1978. **10**(6): p. 539-544.
61. Zhan, Y., et al., *Determination of Free and Deconjugated Testosterone and Epitestosterone in Urine Using SPME and LC–MS/MS.* Bioanalysis., 2011. **3**(1): p. 23-30.

62. Magnuson, B.A., et al., *Biological fate of low-calorie sweeteners.* Nutrition reviews, 2016. **74**(11): p. 670-689.
63. Johnson, C.H., et al., *Novel metabolites and roles for α-tocopherol in humans and mice discovered by mass spectrometry–based metabolomics.* The American journal of clinical nutrition, 2012. **96**(4): p. 818-830.
64. Adlercreutz, H., et al., *Lignan and isoflavonoid conjugates in human urine.* The Journal of steroid biochemistry and molecular biology, 1995. **52**(1): p. 97-103.
65. Feliciano, R.P., et al., *Identification and quantification of novel cranberry-derived plasma and urinary (poly)phenols.* Archives of biochemistry and biophysics, 2016. **599**: p. 31-41.
66. Jo, J.H., S.J. Kim, and S. Lee, *Investigation of the regulatory effects of saccharin on cytochrome P450s in male ICR mice.* The FASEB journal, 2016. **30**(S1).
67. Grice✠, H.C. and L.A. Goldsmith, *Sucralose—an overview of the toxicity data.* Food and chemical toxicology, 2000. **38**: p. 1-6.
68. Dorantes, A. and S. Stavchansky, *Pharmacokinetic and metabolic disposition of p-chloro-m-xylenol (PCMX) in dogs.* Pharmaceutical research, 1992. **9**(5): p. 677-682.
69. Abbas, S., et al., *Metabolism of Parabens (4-Hydroxybenzoic Acid Esters) by Hepatic Esterases and UDP-Glucuronosyltransferases in Man.* DRUG METABOLISM AND PHARMACOKINETICS, 2010. **25**(6): p. 568-577.
70. Gewurtz, S.B., et al., *Influence of Conjugation on the Fate of Pharmaceuticals and Hormones in Canadian Wastewater Treatment Plants.* ACS ES&T water, 2022. **2**(2): p. 329-338.
71. Othman, A.A., et al., *A method for improved detection of 8-isoprostaglandin F2α/β and benzodiazepines in wastewater.* The Science of the total environment, 2022. **851**: p. 158061-158061.
72. Bowers, I. and B. Subedi, *Isoprostanes in wastewater as biomarkers of oxidative stress during COVID-19 pandemic.* Chemosphere (Oxford), 2021. **271**: p. 129489.
73. Ryu, Y., et al., *Increased levels of the oxidative stress biomarker 8-iso-prostaglandin F 2α in wastewater associated with tobacco use.* Scientific reports, 2016. **6**: p. 39055.
74. Rodríguez-Álvarez, T., et al., *Alcohol and cocaine co-consumption in two European cities assessed by wastewater analysis.* The Science of the total environment, 2015. **536**: p. 91-98.
75. Cimino, C.O., et al., *An LC–MS method to determine concentrations of isoflavones and their sulfate and glucuronide conjugates in urine.* Clinica chimica acta, 1999. **287**(1): p. 69-82.
76. Dwivedi, P., et al., *Impact of enzymatic hydrolysis on the quantification of total urinary concentrations of chemical biomarkers.* Chemosphere (Oxford), 2018. **199**(C): p. 256-262.
77. David, A., et al., *Acetaminophen metabolism revisited using non-targeted analyses: Implications for human biomonitoring.* Environment international, 2021. **149**: p. 106388.
78. Sempio, C., et al., *Optimization of recombinant β-glucuronidase hydrolysis and quantification of eight urinary cannabinoids and metabolites by liquid chromatography tandem mass spectrometry.* Drug testing and analysis, 2018. **10**(3): p. 518-529.
79. Sims, N., J. Rice, and B. Kasprzyk-Hordern, *An ultra-high-performance liquid chromatography tandem mass spectrometry method for oxidative stress biomarker analysis in wastewater.* Analytical and bioanalytical chemistry, 2019. **411**(11): p. 2261-2271.
